# Supplementary material for: Cohort studies on 71 outcomes among people with atopic eczema in UK primary care data
Source: Nat Commun. 2024 Nov 6;15:9573. doi: 10.1038/s41467-024-54035-1 (PMC11541564; doi:10.1038/s41467-024-54035-1)
Supplement: Supplementary file 1 — Supplementary Information [file 41467_2024_54035_MOESM1_ESM.pdf]

Supplementary information for "Cohort studies on 71 outcomes among people with atopic eczema in UK primary care data"

Supplementary Notes 1: Results by category

Atopic and allergic diseases

Of all outcomes, eczema was most strongly associated (i.e., the largest adjusted hazard ratio) with **food allergy** (adjusted HR [aHR] 4.03 [3.95-4.11]; rate difference per 1,000 person-years [RD] 1.48). There were strong associations with **allergic conjunctivitis** (aHR 2.01 [1.98-2.04]; RD 1.1), **asthma** (aHR 1.87 [1.86-1.89]; RD 5.4), and **allergic rhinitis** (aHR 1.92 [1.90-1.93]; RD 5.3), with asthma and allergic rhinitis having the largest rate differences of all outcomes. The was also a strong association with **eosinophilic oesophagitis**, however with wider confidence intervals and a small rate difference (aHR 1.64 [1.42-1.90]; RD 0.0).

Effect sizes decreased with cohort age cut-off for food allergy (<18: 5.30 [5.18-5.43]; any age: 4.03 [3.95-4.11]; 18+: 2.00 [1.94-2.07]; 40+: 1.64 [1.56-1.72]), asthma (<18: 2.11 [2.09-2.13]; any age: 1.87 [1.86-1.89]; 18+: 1.56 [1.54-1.58]; 40+: 1.44 [1.42-1.46]), and allergic rhinitis (<18: 2.11 [2.09-2.13]; any age: 1.92 [1.90-1.93]; 18+: 1.64 [1.63-1.66]; 40+: 1.57 [1.55-1.59]). For all outcomes in this category HRs were attenuated in analyses excluding non-consulters (e.g., for food allergy 3.29 [3.17-3.41]), however evidence for associations remained strong.

Evidence for a dose-response relationship with eczema severity was stronger for food allergy (e.g., additionally hospital-admission-adjusted aHR mild 3.66 [3.59-3.75]; mod. 4.19 [4.06-4.32]; sev. 5.72 [4.81-6.80]) and allergic conjunctivitis (mild 1.80 [1.77-1.83]; mod. 2.16 [2.12-2.21]; sev. 2.43 [2.14-2.77]), than it was for asthma (mild 1.75 [1.74-1.77]; mod. 1.84 [1.82-1.86]; sev. 1.87 [1.75-2.00]), allergic rhinitis (mild 1.82 [1.80-1.83]; mod. 1.94 [1.92-1.96]; sev. 1.93 [1.80-2.07]) and eosinophilic oesophagitis (where CIs overlapped the null).

Immune mediated diseases

Eczema was strongly associated with **alopecia areata** (aHR 1.77 [1.71-1.83]; RD 0.18) and **urticaria** (aHR 1.58 [1.57-1.60]; RD 1.87). There was also an association with **chronic obstructive pulmonary disease (COPD)** (aHR 1.22 [1.20-1.23]; RD 1.15).

Effect sizes increased with cohort age cut-off for alopecia areata (<18: 1.61 [1.53-1.69]; any age: 1.77 [1.71-1.83]; 18+: 1.76 [1.69-1.83]; 40+: 1.87 [1.76-1.99]) and urticaria (<18: 1.38 [1.36-1.40]; any age: 1.58 [1.57-1.60]; 18+: 1.77 [1.75-1.80]; 40+: 1.85 [1.82-1.88]). Effect estimates were also larger when using the more severe cohort instead of the any-age cohort for alopecia areata (2.12 [2.03-2.22]) and urticaria (1.71 [1.69-1.74]). For all outcomes in this category, HRs were attenuated in analyses excluding non-consulters (e.g., for alopecia areata aHR 1.60 [1.51-1.70]), however evidence for associations remained strong.

For alopecia areata and urticaria, HRs were larger in moderate eczema than mild eczema, but not larger in severe eczema than moderate eczema (e.g., for alopecia areata: additionally hospital-admission-adjusted aHR mild 1.45 [1.38-1.51]; mod. 2.16 [2.06-2.26]; sev. 2.18 [1.69-2.81]). For COPD we saw a dose-response relationship with eczema severity (mild 1.11 [1.08-1.13; mod. 1.16 [1.14-1.18]; sev. 1.45 [1.33-1.58]).

Skin infection

Eczema was strongly associated with **molluscum contagiosum** (aHR 1.81 [1.79-1.83]; RD 1.85), **impetigo** (aHR 1.49 [1.48-1.51]; RD 2.08), **herpes simplex** (aHR 1.51 [1.49-1.53]; RD 0.94), **dermatophyte infection** (aHR 1.60 [1.59-1.61]; RD 3.75), and **cutaneous warts** (aHR 1.30 [1.29-1.31]; RD 3.04), with rate differences larger than in any other category except atopic and allergic conditions.

While effect estimates were attenuated when using the older cohorts instead of the any age cohort for molluscum contagiosum (18+ aHR 1.56 [1.47-1.65]; 40+ aHR 1.65 [1.50-1.82]), they were increased for impetigo (18+ aHR 1.67 [1.64-1.70]; 40+ aHR 1.70 [1.66-1.75]) and were not considerably changed for herpes simplex and cutaneous warts. Effect estimates were attenuated for dermatophyte infection when using the <18 cohort (aHR 1.45 [1.43-1.46]). For all outcomes in this category HRs were attenuated in analyses excluding non-consulters (e.g., for molluscum contagiosum 1.69 [1.64-1.74]), however evidence for associations remained strong.

Evidence for dose response relationships with eczema severity was stronger for molluscum contagiosum (additionally hospital-admission-adjusted aHR mild 1.76 [1.74-1.78]; mod. 2.01 [1.96-2.06]; sev. 2.17 [1.84-2.55]), impetigo and herpes simplex than it was for dermatophyte infection (mild 1.49 [1.47-1.50]; mod. 1.62 [1.61-1.64]; sev. 1.41 [1.33-1.50]) and cutaneous warts.

Liver diseases

Eczema was associated with **autoimmune liver disease** (aHR 1.32 [1.21-1.43]; RD 0.01), **fibrosis/sclerosis/cirrhosis** (aHR 1.26 [1.21-1.32]; RD 0.09), **fatty liver** (aHR 1.24 [1.22-1.26]; RD 0.28), **oesophageal varices** (aHR 1.19 [1.10-1.30]; RD 0.02), and to a lesser extent with **cholecystitis** (aHR 1.10 [1.07-1.14]; RD 0.05), with relatively low rate differences for all outcomes in this category.

Effect estimates were somewhat increased when using the more severe cohort for autoimmune liver disease (aHR 1.40 [1.27-1.56]), fibrosis/sclerosis/cirrhosis (aHR 1.33 [1.27-1.40]) and oesophageal varices (aHR 1.38 [1.25-1.52]). For all outcomes in this category HRs were attenuated in analyses excluding non-consulters; while for fibrosis/sclerosis/cirrhosis there was only a small attenuation (aHR 1.23 [1.16-1.31]), there were larger attenuations for autoimmune liver disease (1.20 [1.07-1.34]) and fatty liver (1.14 [1.11-1.16]), and for oesophageal varices and cholecystitis the lower bounds of the 99% confidence intervals were at, or close to, the null.

Effect estimates were considerably larger in severe eczema than mild or moderate eczema for autoimmune liver disease (additionally hospital-admission-adjusted aHR mild 1.02 [0.89-1.16]; mod. 1.14 [1.02-1.27]; sev. 5.32 [3.46-8.18]), fatty liver, fibrosis/sclerosis/cirrhosis, and oesophageal varices, but not for cholecystitis (mild 0.93 [0.89-0.97]; mod. 0.96 [0.93-0.99]; sev. 1.09 [0.93-1.28]).

Digestive system – inflammatory bowel diseases

Eczema was strongly associated with **Crohn's disease** (aHR 1.62 [1.54-1.69]; RD 0.09), and **ulcerative colitis** (aHR 1.40 [1.34-1.46]; RD 0.08).

For Crohn's disease, but not for ulcerative colitis, using the other cohorts changed estimates, however confidence intervals from different cohorts overlapped (any age aHR 1.62 [1.54-1.69]; 18+ aHR 1.54 [1.46-1.61]; 40+ aHR 1.41 [1.32-1.50]; <18 aHR 1.82 [1.67-1.97]; more severe aHR 1.64 [1.55-1.74]). For both outcomes HRs were attenuated in analyses excluding non-consulters (e.g., for Crohn's disease 1.38 [1.28-1.48]), however evidence for associations remained strong.

Effect estimates were considerably larger in severe eczema than mild or moderate eczema for both Crohn's disease (additionally hospital-admission-adjusted aHR mild 1.14 [1.06-1.22]; mod. 1.29 [1.20-1.38]; sev. 4.92 [3.74-6.47]) and ulcerative colitis (mild 1.12 [1.06-1.19]; mod. 1.19 [1.12-1.25]; sev. 2.62 [2.08-3.31]).

Digestive system – diseases of the oesophagus

Eczema was associated with **oesophagitis** (aHR 1.25 [1.23-1.27]; RD 0.48), **gastro oesophageal reflux** (aHR 1.25 [1.24-1.26]; RD 1.10), and to a lesser extent with **Barrett's oesophagus** (aHR 1.16 [1.12-1.20]; RD 0.08).

Using the other cohorts caused little change in effect estimates, except an attenuation in the <18 cohort for oesophagitis (1.16 [1.13-1.19]). HRs were attenuated when excluding non-consulters.

There was some evidence for a dose-response relationship with eczema severity for oesophagitis (additionally hospital-admission-adjusted aHR mild 1.11 [1.09-1.13]; mod. 1.14 [1.12-1.16]; sev. 1.26 [1.15-1.37]), but not gastro oesophageal reflux or Barrett's oesophagus.

Digestive system – other

Eczema was strongly associated with **coeliac disease** (aHR 1.42 [1.37-1.47]; RD 0.10) and **irritable bowel syndrome** (aHR 1.31 [1.29-1.32]; RD 0.67). Eczema was also associated with **gastritis and duodenitis** (aHR 1.21 [1.20-1.23]; RD 0.60), and to a lesser extent with **diverticular disease** (aHR 1.17 [1.15-1.18]; RD 0.64), **peptic ulcer disease** (aHR 1.16 [1.13-1.19]; RD 0.08), **abdominal hernia** (aHR 1.13 [1.12-1.15]; RD 0.30) and **pancreatitis** (aHR 1.11 [1.07-1.15]; RD 0.03). Eczema was only weakly associated with **peritonitis** (aHR 1.07 [1.00-1.13]; RD 0.01) and **appendicitis** (aHR 1.07 [1.05-1.09]; RD 0.06).

Using the other cohorts caused little change in effect estimates, except attenuated HRs in the <18 cohort for coeliac disease (1.34 [1.26-1.42]), irritable bowel syndrome (1.22 [1.19-1.25]), and abdominal hernia (1.05 [1.01-1.09]). For all outcomes in this category HRs were attenuated in analyses excluding non-consulters; while for some outcomes evidence remained strong (e.g., coeliac disease aHR 1.33 [1.25-1.40]), for pancreatitis, appendicitis, and peritonitis the 99% confidence intervals overlapped the null.

For coeliac disease, we found evidence of stronger associations with moderate eczema but not with severe eczema, be it in comorbidity-adjusted (mild 1.31 [1.25-1.37]; mod. 1.55 [1.47-1.63]; sev. 1.42 [1.07-1.90]) or additionally hospital-admission-adjusted results (mild 1.17 [1.11-1.23]; mod. 1.36 [1.29-1.44]; sev. 1.01 [0.74-1.38]). We found evidence of a dose-response relationship with eczema severity for gastritis and duodenitis (additionally hospital-admission-adjusted aHR mild 1.07 [1.05-1.08]; mod. 1.11 [1.09-1.13]; sev. 1.39

[1.29-1.49]) and diverticular disease, but not for irritable bowel syndrome, pancreatitis, and appendicitis.

Mental health disorders and substance use

Eczema was associated with **anxiety** (aHR 1.17 [1.16-1.17]; RD 1.88) and **depression** (aHR 1.17 [1.16-1.17]; RD 1.96) with rate differences on par with skin infections and larger than those from any other category other than atopic and allergic conditions. Eczema was also associated, however with a much lower rate difference, with **alcohol abuse** (aHR 1.13 [1.10-1.15]; RD 0.13). Eczema was also associated to a lesser extent with **cigarette smoking** (aHR 1.11 [1.11-1.12]; RD 3.66), with a large rate difference.

Effect estimates were not considerably changed when using the any-age, 40+ or more severe cohorts instead of the 18+ cohort for any of the outcomes in this category. However, using the <18 cohort led to attenuated HRs for depression and alcohol abuse (aHR 1.00 [0.92-1.08]), but an increased HR for cigarette smoking (aHR 1.20 [1.19-1.22]). HRs were attenuated when excluding non-consulters, especially for cigarette smoking (aHR 1.03 [1.02-1.04]).

While we found evidence for a dose-response relationship with eczema severity for depression (additionally hospital-admission-adjusted aHR mild 1.08 [1.07-1.09]; mod. 1.12 [1.11-1.13]; sev. 1.25 [1.19-1.31]), we did not find this for anxiety (mild 1.11 [1.11-1.12]; mod. 1.13 [1.12-1.14]; sev. 1.04 [0.99-1.09]), alcohol abuse or cigarette smoking.

Cardiovascular diseases

Eczema was associated with **thromboembolic diseases** (aHR 1.25 [1.23-1.27]; RD 0.51), and to a lesser extent with **peripheral artery disease** (aHR 1.18 [1.16-1.21]; RD 0.21), **heart failure** (aHR 1.16 [1.15-1.18]; RD 0.52), **coronary artery disease** (aHR 1.15 [1.14-1.16]; RD 1.07), **hypertension** (aHR 1.11 [1.10-1.12]; RD 1.40), **myocardial infarction** (aHR 1.09 [1.07-1.11]; RD 0.15) and **stroke** (aHR 1.09 [1.07-1.10]; RD 0.27).

Using the other cohorts instead of the 18+ cohort did not change effect estimates considerably, except an attenuated HR in the <18 cohort for thromboembolic diseases (aHR 1.08 [1.01-1.16]). For all outcomes in this category HRs were attenuated in analyses excluding non-consulters; while evidence of association remained relatively strong for thromboembolic diseases (aHR 1.18 [1.15-1.20]), HRs were close to the null for coronary artery disease (aHR 1.04 [1.02-1.05]), hypertension, myocardial infarction, and stroke.

For all outcomes in this category there was evidence of a dose-response relationship with eczema severity; the largest HR for severe eczema being for thromboembolic diseases (additionally hospital-admission-adjusted aHR mild 1.04 [1.01-1.07]; mod. 1.17 [1.15-1.19]; sev. 1.67 [1.53-1.83]) and the smallest for coronary artery disease (mild 1.07 [1.06-1.09]; mod. 1.08 [1.06-1.09]; sev. 1.14 [1.07-1.21]).

Metabolic diseases

Eczema was associated with **obesity** (aHR 1.22 [1.21-1.23]; RD 0.77), and to a lesser extent with **dyslipidaemia** (aHR 1.13 [1.12-1.14]; RD 0.50), **diabetes mellitus** (aHR 1.13 [1.12-1.14]; RD 0.45) and **metabolic syndrome** (aHR 1.25 [1.10-1.42]; RD 0.01).

Using the other cohorts instead of the any-age cohorts caused little change in effect estimates, except for a HR attenuated to the null for diabetes in the <18 cohort (1.03 [0.98-1.08]). HRs were attenuated after excluding non-consulters, and for metabolic syndrome and dyslipidaemia were at, or close to, the null.

We found some evidence of a dose-response relationship with eczema severity for diabetes (additionally hospital-admission-adjusted aHR mild 1.03 [1.01-1.05]; mod. 1.11 [1.09-1.12]; sev. 1.18 [1.11-1.27]), but not for the other outcomes in this category.

Neurological diseases

Eczema was associated with **peripheral neuropathies** (aHR 1.21 [1.20-1.22]; RD 2.13) with a considerable rate difference similar to that of allergic rhinitis. Eczema was also associated to **migraine** (aHR 1.18 [1.17-1.19]; RD 0.66), and to a lesser extent to **epilepsy** (aHR 1.15 [1.12-1.18]; RD 0.09) and **multiple sclerosis** (aHR 1.11 [1.03-1.18]; RD 0.01). Eczema was not, or only very weakly, associated with **Alzheimer's dementia** (aHR 1.04 [1.02-1.06]; RD 0.07), **vascular dementia** (aHR 1.07 [1.04-1.10]; RD 0.07) and **Parkinson's disease** (aHR 1.02 [0.98-1.05]; RD 0.01).

Using the other cohorts caused little change in effect estimates, except attenuations when using the <18 cohorts for peripheral neuropathies (aHR 1.08 [1.05-1.11]) and epilepsy (aHR 1.04 [1.01-1.08]). Excluding non-consulters attenuated HRs for all outcomes, including to the null for multiple sclerosis (0.99 [0.90-1.10]).

We found some evidence for a dose-response relationship with eczema severity for peripheral neuropathies (additionally hospital-admission-adjusted aHR mild 1.12 [1.11-1.14]; mod. 1.17 [1.16-1.18]; sev. 1.20 [1.14-1.27]) but not for the other outcomes in this category.

ADHD and autism

Eczema was weakly associated with **ADHD** (aHR 1.08 [1.06-1.11]; RD 0.06) and **autism** (aHR 1.15 [1.13-1.17]; RD 0.15).

While using any of the other cohorts instead of the any-age cohort for ADHD caused little change in the effect estimates and widened confidence intervals to include the null, for autism, using the 40+ cohort instead of the any-age cohort considerably increased the effect estimate (aHR 1.59 [1.45-1.76]).

Effect estimates were similar for mild, moderate and severe eczema, with wide CIs for severe eczema.

Bone health

Eczema was associated with **osteoporosis** (aHR 1.18 [1.16-1.20]; RD 0.53), and to a lesser extent (in order of decreasing aHR) with **spine fracture** (aHR 1.17 [1.14-1.20]; RD 0.13), **pelvis fracture** (aHR 1.11 [1.07-1.15]; RD 0.05), **hip fracture** (aHR 1.11 [1.09-1.13]; RD 0.14), and **wrist fracture** (aHR 1.07 [1.05-1.08]; RD 0.17).

Using the other cohorts, instead of the 18+ cohort caused little change in effect estimates, with wide CIs in the <18 cohort. Excluding non-consulters somewhat attenuated HRs (e.g., for osteoporosis aHR 1.12 [1.10-1.14]).

We found relatively large HRs for severe eczema for osteoporosis (additionally hospital-admission-adjusted aHR mild 1.05 [1.03-1.07]; mod. 1.07 [1.05-1.09]; sev. 2.70 [2.52-2.90]) and all fractures, except for wrist fracture (mild 1.01 [0.99-1.03] ; mod. 1.03 [1.01-1.05]; sev. 1.08 [0.98-1.20]).

Cancer

Eczema was not, or only weakly, associated with solid organ cancers including **lung cancer** (aHR 1.05 [1.02-1.08]; RD 0.04), **breast cancer** (aHR 1.03 [1.01-1.05]; RD 0.04), **prostate cancer** (aHR 1.01 [0.99-1.03]; RD 0.02), **pancreatic cancer** (aHR 1.05 [1.00-1.11]; RD 0.01), **central nervous system cancers** (aHR 1.05 [1.01-1.10]; RD 0.01), and **melanoma** (aHR 1.08 [1.05-1.11]; RD 0.03). Eczema was associated with **myeloma** (aHR 1.10 [1.03-1.18]; RD 0.01), and **non-melanoma skin cancer** (aHR 1.13 [1.11-1.14]; RD 0.25), non-melanoma skin cancer having the largest rate difference of any cancer, however still lower than many other outcomes. Eczema was associated with **non-Hodgkin's lymphoma** (aHR 1.26 [1.21-1.32]; RD 0.05) and strongly associated with **Hodgkin lymphoma** (aHR 1.85 [1.66-2.06]; RD 0.02), however with very small rate differences for both outcomes.

Using the other cohorts caused little change in effect estimates. Excluding non-consulters attenuated effect estimates; CIs for central nervous system cancers, myeloma and melanoma now crossed the null, while evidence for an association with lymphomas and nonmelanoma skin cancer remained.

Evidence for a dose-response relationship with eczema severity was found for non-Hodgkin lymphoma and nonmelanoma skin cancer. For Hodgkin lymphoma we found increasing HRs, but wide CIs for severe eczema (additionally hospital-admission-adjusted aHR mild 1.13 [0.95-1.35] ; mod. 1.58 [1.37-1.82]; sev. 2.02 [1.03-3.98]).

Supplementary Table 1: Eczema compared to no eczema

Outcome	Main cohort	events	person-years	Crude Rate (per 1,000 person-years) exposed	unexposed	Crude Rate difference	Estimated Rate difference[2]	Hazard ratio (99% confidence interval)[1] minimally-adjusted	comorbidity-adjusted	drug-adjusted
**Atopic and allergic**										
Food allergy	any age	94,867	129,677,918	2.0	0.4	1.53	1.48	4.37 [4.29-4.45]*	4.03 [3.95-4.11]*	4.01 [3.93-4.09]*
Allergic Conjunctivitis	any age	154,539	128,319,292	2.2	1.0	1.22	1.10	2.21 [2.18-2.25]*	2.01 [1.98-2.04]*	2.00 [1.97-2.03]*
Allergic Rhinitis	any age	699,425	109,630,416	11.1	5.2	5.93	5.32	2.09 [2.07-2.10]*	1.92 [1.90-1.93]*	1.90 [1.89-1.92]*
Asthma	any age	699,010	99,106,832	11.5	5.9	5.57	5.35	1.97 [1.95-1.98]*	1.87 [1.86-1.89]*	1.83 [1.82-1.85]*
Eosinophilic Oesophagitis	any age	2,208	132,363,302	0.0	0.0	0.01	0.01	1.96 [1.72-2.23]*	1.64 [1.42-1.90]*	1.62 [1.40-1.88]*
**Cancer**										
Hodgkin lymphoma	any age	3,522	132,300,421	0.0	0.0	0.02	0.02	1.88 [1.69-2.08]*	1.85 [1.66-2.06]*	1.84 [1.65-2.05]*
Non-Hodgkin lymphoma	any age	25,915	132,075,634	0.2	0.2	0.03	0.05	1.30 [1.25-1.36]*	1.26 [1.21-1.32]*	1.26 [1.21-1.31]*
Nonmelanoma skin cancer	any age	267,492	128,656,533	2.2	2.0	0.16	0.25	1.17 [1.16-1.19]*	1.13 [1.11-1.14]*	1.13 [1.11-1.14]*
Myeloma	any age	10,458	132,301,340	0.1	0.1	0.00	0.01	1.13 [1.06-1.21]*	1.10 [1.03-1.18]*	1.10 [1.02-1.18]*
Melanoma	any age	55,580	131,347,273	0.4	0.4	0.00	0.03	1.11 [1.08-1.14]*	1.08 [1.05-1.11]*	1.08 [1.05-1.12]*
CNS cancers	any age	27,380	132,126,472	0.2	0.2	0.00	0.01	1.10 [1.06-1.15]*	1.05 [1.01-1.10]	1.05 [1.01-1.10]
Pancreatic cancer	18+	16,298	92,340,078	0.2	0.2	0.02	0.01	1.09 [1.03-1.15]*	1.05 [1.00-1.11]	1.05 [0.99-1.11]
Lung cancer	18+	73,294	92,155,590	0.9	0.8	0.09	0.04	1.13 [1.10-1.15]*	1.05 [1.02-1.08]*	1.05 [1.02-1.08]*
Breast cancer	18+	103,962	90,553,313	1.2	1.1	0.07	0.04	1.04 [1.02-1.06]*	1.03 [1.01-1.05]*	1.03 [1.01-1.06]*
Prostate cancer	40+	101,527	58,336,375	1.8	1.7	0.12	0.02	1.03 [1.01-1.05]*	1.01 [0.99-1.03]	1.01 [0.99-1.03]
**Skin infection**										
Molluscum contagiosum	any age	306,757	126,603,018	4.1	2.0	2.13	1.85	1.84 [1.82-1.86]*	1.81 [1.79-1.83]*	1.81 [1.79-1.83]*
Dermatophyte infection	any age	774,374	112,045,992	10.0	6.1	3.88	3.75	1.67 [1.66-1.69]*	1.60 [1.59-1.61]*	1.60 [1.58-1.61]*
Herpes simplex	any age	240,103	126,651,269	2.8	1.7	1.09	0.94	1.66 [1.64-1.68]*	1.51 [1.49-1.53]*	1.51 [1.49-1.53]*
Impetigo	any age	513,128	118,822,044	6.3	3.8	2.45	2.08	1.55 [1.54-1.56]*	1.49 [1.48-1.51]*	1.49 [1.48-1.50]*
Cutaneous warts	any age	1,102,269	109,295,803	13.2	9.3	3.85	3.04	1.35 [1.35-1.36]*	1.30 [1.29-1.31]*	1.30 [1.29-1.31]*
**Immune mediated**										
Alopecia Areata	any age	34,376	131,681,350	0.4	0.2	0.20	0.18	1.87 [1.81-1.93]*	1.77 [1.71-1.83]*	1.76 [1.70-1.82]*
Urticaria	any age	414,078	122,363,873	5.1	3.0	2.10	1.87	1.68 [1.67-1.70]*	1.58 [1.57-1.60]*	1.58 [1.56-1.59]*
COPD	40+	269,930	54,706,063	6.5	4.6	1.94	1.15	1.41 [1.40-1.43]*	1.22 [1.20-1.23]*	1.18 [1.17-1.20]*
**Digestive system**										

Outcome	Main cohort	events	person-years	Crude Rate (per 1,000 person-years)		Crude Rate difference	Estimated Rate difference[2]	Hazard ratio (99% confidence interval)[1]		
				exposed	unexposed			minimally-adjusted	comorbidity-adjusted	drug-adjusted
Crohn's disease	any age	20,230	131,801,877	0.2	0.1	0.10	0.09	1.75 [1.68-1.83]*	1.62 [1.54-1.69]*	1.59 [1.51-1.66]*
Coeliac disease	any age	31,782	131,778,936	0.3	0.2	0.11	0.10	1.52 [1.46-1.57]*	1.42 [1.37-1.47]*	1.42 [1.36-1.47]*
Ulcerative colitis	any age	27,027	131,570,373	0.3	0.2	0.08	0.08	1.49 [1.43-1.55]*	1.40 [1.34-1.46]*	1.37 [1.31-1.43]*
Irritable bowel syndrome	any age	272,010	123,906,183	2.8	2.0	0.82	0.67	1.47 [1.45-1.48]*	1.31 [1.29-1.32]*	1.31 [1.29-1.32]*
Oesophagitis	any age	248,927	125,676,829	2.4	1.9	0.52	0.48	1.40 [1.38-1.41]*	1.25 [1.23-1.27]*	1.24 [1.23-1.26]*
Gastro oesophageal reflux	any age	539,815	120,056,979	5.5	4.2	1.31	1.10	1.39 [1.38-1.40]*	1.25 [1.24-1.26]*	1.24 [1.23-1.25]*
Gastritis and duodenitis	any age	356,381	126,112,608	3.4	2.7	0.75	0.60	1.35 [1.33-1.36]*	1.21 [1.20-1.23]*	1.21 [1.19-1.22]*
Diverticular disease	18+	314,741	87,721,438	4.4	3.4	1.02	0.64	1.29 [1.28-1.31]*	1.17 [1.15-1.18]*	1.16 [1.15-1.18]*
Barrett’s oesophagus	18+	42,753	91,753,003	0.6	0.4	0.11	0.08	1.27 [1.23-1.31]*	1.16 [1.12-1.20]*	1.16 [1.12-1.20]*
Peptic ulcer disease	any age	71,432	130,748,986	0.6	0.5	0.07	0.08	1.25 [1.22-1.28]*	1.16 [1.13-1.19]*	1.15 [1.12-1.18]*
Abdominal hernia	any age	291,201	125,008,052	2.6	2.3	0.29	0.30	1.20 [1.19-1.22]*	1.13 [1.12-1.15]*	1.13 [1.11-1.14]*
Pancreatitis	any age	37,452	131,799,502	0.3	0.3	0.04	0.03	1.23 [1.18-1.27]*	1.11 [1.07-1.15]*	1.10 [1.06-1.14]*
Appendicitis	any age	109,685	129,865,984	1.0	0.8	0.14	0.06	1.14 [1.11-1.16]*	1.07 [1.05-1.09]*	1.07 [1.05-1.09]*
Peritonitis	any age	13,032	132,170,298	0.1	0.1	0.01	0.01	1.15 [1.09-1.22]*	1.07 [1.00-1.13]	1.06 [1.00-1.13]
**Liver**										
Autoimmune liver disease	any age	6,352	132,269,823	0.1	0.0	0.02	0.01	1.44 [1.33-1.56]*	1.32 [1.21-1.43]*	1.31 [1.20-1.42]*
Fibrosis/sclerosis/cirrhosis	18+	29,160	92,025,270	0.4	0.3	0.13	0.09	1.43 [1.38-1.49]*	1.26 [1.21-1.32]*	1.26 [1.20-1.31]*
Fatty liver	any age	148,815	131,108,036	1.4	1.1	0.36	0.28	1.43 [1.41-1.46]*	1.24 [1.22-1.26]*	1.24 [1.21-1.26]*
Oesophageal varices	18+	8,813	92,294,383	0.1	0.1	0.04	0.02	1.43 [1.33-1.53]*	1.19 [1.10-1.30]*	1.19 [1.09-1.29]*
Cholecystitis	any age	66,438	131,413,446	0.6	0.5	0.06	0.05	1.22 [1.19-1.25]*	1.10 [1.07-1.14]*	1.10 [1.07-1.13]*
**Cardiovascular**										
Thromboembolic diseases	18+	177,337	89,591,235	2.5	1.9	0.66	0.51	1.35 [1.33-1.37]*	1.25 [1.23-1.27]*	1.24 [1.22-1.26]*
Peripheral artery disease	18+	103,675	90,508,776	1.4	1.1	0.31	0.21	1.29 [1.26-1.32]*	1.18 [1.16-1.21]*	1.18 [1.15-1.20]*
Heart failure	18+	270,404	89,987,240	3.7	2.8	0.85	0.52	1.27 [1.25-1.28]*	1.16 [1.15-1.18]*	1.15 [1.14-1.17]*
Coronary artery disease	18+	540,912	77,641,301	8.3	6.7	1.61	1.07	1.24 [1.23-1.25]*	1.15 [1.14-1.16]*	1.15 [1.13-1.16]*
Hypertension	18+	775,356	67,565,647	14.0	10.9	3.16	1.40	1.17 [1.16-1.17]*	1.11 [1.10-1.12]*	1.11 [1.10-1.12]*
Stroke	18+	261,037	89,936,081	3.4	2.8	0.56	0.27	1.18 [1.16-1.19]*	1.09 [1.07-1.10]*	1.09 [1.07-1.10]*
Myocardial infarction	18+	151,717	89,598,403	1.9	1.6	0.29	0.15	1.17 [1.15-1.19]*	1.09 [1.07-1.11]*	1.08 [1.06-1.10]*
**Metabolic**										
Metabolic syndrome	any age	3,040	132,305,044	0.0	0.0	0.01	0.01	1.45 [1.29-1.63]*	1.25 [1.10-1.42]*	1.25 [1.10-1.42]*

Outcome	Main cohort	events	person-years	Crude Rate (per 1,000 person-years) exposed	unexposed	Crude Rate difference	Estimated Rate difference[2]	Hazard ratio (99% confidence interval)[1] minimally-adjusted	comorbidity-adjusted	drug-adjusted
Obesity	any age	441,442	122,842,347	4.3	3.4	0.88	0.77	1.35 [1.33-1.36]*	1.22 [1.21-1.23]*	1.21 [1.20-1.22]*
Dyslipidaemia	any age	472,695	120,777,185	4.4	3.8	0.55	0.50	1.22 [1.20-1.23]*	1.13 [1.12-1.14]*	1.13 [1.12-1.14]*
Diabetes mellitus	any age	424,701	122,434,156	3.9	3.4	0.56	0.45	1.23 [1.22-1.24]*	1.13 [1.12-1.14]*	1.12 [1.11-1.13]*
**Neurological**										
Peripheral neuropathies	18+	735,314	75,941,901	12.3	9.1	3.20	2.13	1.33 [1.32-1.34]*	1.21 [1.20-1.22]*	1.20 [1.19-1.21]*
Migraine	any age	433,361	122,281,709	4.4	3.3	1.03	0.66	1.28 [1.27-1.30]*	1.18 [1.17-1.19]*	1.17 [1.16-1.19]*
Epilepsy	any age	79,458	129,897,303	0.7	0.6	0.12	0.09	1.22 [1.19-1.24]*	1.15 [1.12-1.18]*	1.15 [1.12-1.18]*
Multiple sclerosis	18+	10,608	91,991,931	0.1	0.1	0.02	0.01	1.16 [1.08-1.23]*	1.11 [1.03-1.18]*	1.09 [1.02-1.17]
Vascular dementia	40+	57,217	59,173,934	1.1	0.9	0.18	0.07	1.13 [1.10-1.16]*	1.07 [1.04-1.10]*	1.07 [1.04-1.10]*
Alzheimer's dementia	40+	101,271	58,896,196	1.9	1.7	0.24	0.07	1.06 [1.04-1.09]*	1.04 [1.02-1.06]*	1.04 [1.02-1.07]*
Parkinson's disease	40+	40,973	59,007,426	0.7	0.7	0.06	0.01	1.05 [1.02-1.09]*	1.02 [0.98-1.05]	1.02 [0.98-1.06]
**Bone health**										
Osteoporosis	18+	249,956	88,699,650	3.5	2.7	0.79	0.53	1.26 [1.24-1.27]*	1.18 [1.16-1.20]*	1.17 [1.16-1.19]*
Spine fracture	18+	67,844	91,764,889	0.9	0.7	0.21	0.13	1.28 [1.24-1.31]*	1.17 [1.14-1.20]*	1.16 [1.13-1.20]*
Pelvis fracture	18+	39,320	91,998,149	0.5	0.4	0.08	0.05	1.18 [1.14-1.22]*	1.11 [1.07-1.15]*	1.10 [1.06-1.15]*
Hip fracture	18+	114,345	91,558,912	1.4	1.2	0.22	0.14	1.14 [1.12-1.16]*	1.11 [1.09-1.13]*	1.11 [1.08-1.13]*
Wrist fracture	18+	209,406	86,928,363	2.7	2.3	0.35	0.17	1.14 [1.12-1.16]*	1.07 [1.05-1.08]*	1.06 [1.05-1.08]*
**Mental health and substance use**										
Depression	any age	1,173,853	101,880,802	13.7	10.9	2.77	1.96	1.27 [1.26-1.28]*	1.17 [1.16-1.17]*	1.16 [1.15-1.17]*
Anxiety	any age	1,192,074	110,373,722	13.2	10.2	2.97	1.88	1.28 [1.27-1.29]*	1.17 [1.16-1.17]*	1.16 [1.16-1.17]*
Alcohol abuse	18+	85,997	90,151,061	1.1	0.9	0.24	0.13	1.27 [1.24-1.30]*	1.13 [1.10-1.15]*	1.12 [1.10-1.15]*
Cigarette smoking	18+	1,152,377	36,461,715	35.9	30.4	5.51	3.66	1.21 [1.20-1.21]*	1.11 [1.11-1.12]*	1.11 [1.10-1.12]*
**ADHD and autism**										
Autism	any age	117,398	131,252,965	1.2	0.8	0.36	0.15	1.20 [1.17-1.22]*	1.15 [1.13-1.17]*	1.15 [1.13-1.17]*
ADHD	any age	82,899	131,446,061	0.8	0.6	0.23	0.06	1.17 [1.14-1.20]*	1.08 [1.06-1.11]*	1.08 [1.05-1.10]*

[1] Hazard ratios (99% confidence intervals) estimated from Cox models comparing people with eczema to those without eczema. * indicates that the result is significant under Bonferroni-correction.

[2] Rate difference calculated using the estimated rate in the unexposed (rate in the exposed * (1/hazard ratio))

Supplementary Table 2: Eczema compared to no eczema in cohort excluding non-consulters

Outcome	Main cohort	events	person-years	Crude Rate (per 1,000 person-years)		Crude Rate difference	Estimated Rate difference[2]	Hazard ratio (99% confidence interval)[1]	
				exposed	unexposed			minimally-adjusted	comorbidity-adjusted
**Atopic and allergic**									
Food allergy	any age	31,379	39,420,872	1.7	0.4	1.25	1.18	3.51 [3.39-3.63]*	3.29 [3.17-3.41]*
Allergic Conjunctivitis	any age	51,670	38,742,604	2.1	1.0	1.09	0.97	2.00 [1.94-2.05]*	1.84 [1.79-1.89]*
Allergic Rhinitis	any age	206,460	32,340,252	9.6	5.1	4.50	3.69	1.75 [1.73-1.78]*	1.63 [1.60-1.65]*
Asthma	any age	205,112	28,641,937	10.0	6.0	3.99	3.61	1.63 [1.61-1.65]*	1.57 [1.55-1.59]*
Eosinophilic Oesophagitis	any age	745	40,235,828	0.0	0.0	0.01	0.01	1.61 [1.29-2.01]*	1.38 [1.06-1.79]
**Skin infection**									
Molluscum contagiosum	any age	49,080	39,356,781	2.0	0.9	1.09	0.83	1.70 [1.66-1.75]*	1.69 [1.64-1.74]*
Dermatophyte infection	any age	277,126	32,615,442	11.2	7.4	3.73	3.65	1.53 [1.51-1.55]*	1.49 [1.47-1.50]*
Impetigo	any age	107,149	37,280,992	4.0	2.4	1.62	1.21	1.48 [1.45-1.51]*	1.43 [1.40-1.46]*
Herpes simplex	any age	87,128	38,033,887	3.0	2.0	0.99	0.78	1.45 [1.42-1.48]*	1.35 [1.32-1.38]*
Cutaneous warts	any age	295,940	32,720,185	10.7	8.4	2.28	1.77	1.23 [1.22-1.25]*	1.20 [1.19-1.21]*
**Cancer**									
Hodgkin lymphoma	any age	1,422	40,209,682	0.0	0.0	0.02	0.02	1.62 [1.39-1.90]*	1.66 [1.40-1.97]*
Non-Hodgkin lymphoma	any age	14,270	40,078,540	0.4	0.4	0.00	0.05	1.19 [1.13-1.25]*	1.17 [1.11-1.23]*
Nonmelanoma skin cancer	any age	153,228	37,993,558	3.8	4.1	−0.31	0.31	1.12 [1.10-1.13]*	1.09 [1.07-1.11]*
Myeloma	any age	6,139	40,196,423	0.1	0.2	−0.02	0.01	1.07 [0.98-1.16]	1.06 [0.97-1.15]
Lung cancer	18+	43,575	36,006,463	1.1	1.2	−0.11	0.05	1.09 [1.05-1.12]*	1.05 [1.01-1.09]*
CNS cancers	any age	14,158	40,117,177	0.3	0.4	−0.04	0.01	1.04 [0.99-1.10]	1.03 [0.97-1.08]
Melanoma	any age	29,584	39,682,351	0.7	0.8	−0.09	0.01	1.04 [1.00-1.08]	1.02 [0.98-1.06]
Pancreatic cancer	18+	9,718	36,118,069	0.2	0.3	−0.03	0.00	1.02 [0.95-1.09]	1.01 [0.95-1.09]
Breast cancer	18+	54,455	35,154,316	1.5	1.6	−0.10	0.01	1.01 [0.98-1.04]	1.01 [0.98-1.03]
Prostate cancer	40+	55,875	25,598,065	2.1	2.2	−0.14	−0.04	0.99 [0.96-1.02]	0.98 [0.95-1.01]
**Immune mediated**									
Alopecia Areata	any age	10,168	40,007,493	0.4	0.2	0.18	0.14	1.67 [1.57-1.77]*	1.60 [1.51-1.70]*
Urticaria	any age	133,971	36,753,066	5.1	3.1	2.09	1.88	1.66 [1.63-1.68]*	1.58 [1.55-1.60]*
COPD	40+	150,633	23,312,919	7.4	6.1	1.27	1.06	1.28 [1.26-1.30]*	1.17 [1.15-1.19]*

Outcome	Main cohort	events	person-years	Crude Rate (per 1,000 person-years) exposed	Crude Rate (per 1,000 person-years) unexposed	Crude Rate difference	Estimated Rate difference[2]	Hazard ratio (99% confidence interval)[1] minimally-adjusted	Hazard ratio (99% confidence interval)[1] comorbidity-adjusted
**Digestive system**									
Crohn's disease	any age	7,942	39,988,139	0.3	0.2	0.08	0.07	1.44 [1.35-1.54]*	1.38 [1.28-1.48]*
Coeliac disease	any age	12,159	39,974,187	0.4	0.3	0.10	0.09	1.38 [1.31-1.46]*	1.33 [1.25-1.40]*
Ulcerative colitis	any age	11,130	39,868,425	0.3	0.3	0.08	0.08	1.38 [1.30-1.46]*	1.33 [1.25-1.41]*
Irritable bowel syndrome	any age	111,225	36,180,739	3.7	2.8	0.83	0.55	1.28 [1.26-1.30]*	1.18 [1.15-1.20]*
Gastro oesophageal reflux	any age	250,909	34,609,586	8.1	6.9	1.22	1.03	1.23 [1.22-1.25]*	1.15 [1.13-1.16]*
Oesophagitis	any age	123,741	36,806,213	3.6	3.2	0.39	0.43	1.22 [1.20-1.24]*	1.13 [1.11-1.16]*
Gastritis and duodenitis	any age	170,219	37,157,455	5.0	4.4	0.53	0.54	1.20 [1.18-1.22]*	1.12 [1.10-1.14]*
Peptic ulcer disease	any age	39,595	39,327,184	1.0	1.0	−0.01	0.10	1.15 [1.11-1.19]*	1.11 [1.07-1.14]*
Diverticular disease	18+	182,307	33,204,593	5.7	5.4	0.29	0.52	1.17 [1.15-1.19]*	1.10 [1.08-1.12]*
Barrett’s oesophagus	18+	23,979	35,770,599	0.7	0.7	0.02	0.06	1.15 [1.10-1.19]*	1.09 [1.05-1.14]*
Abdominal hernia	any age	129,486	37,173,490	3.6	3.4	0.14	0.24	1.12 [1.10-1.14]*	1.07 [1.05-1.09]*
Pancreatitis	any age	19,679	39,927,481	0.5	0.5	−0.01	0.02	1.11 [1.06-1.16]*	1.05 [1.00-1.10]
Peritonitis	any age	6,200	40,139,173	0.2	0.2	0.00	0.00	1.07 [0.99-1.16]	1.02 [0.93-1.11]
Appendicitis	any age	30,646	39,312,823	0.9	0.8	0.10	0.01	1.06 [1.02-1.10]*	1.01 [0.97-1.05]
**Liver**									
Fibrosis/sclerosis/cirrhosis	18+	15,460	35,951,051	0.5	0.4	0.11	0.10	1.30 [1.24-1.37]*	1.23 [1.16-1.31]*
Autoimmune liver disease	any age	3,262	40,181,687	0.1	0.1	0.01	0.01	1.26 [1.13-1.40]*	1.20 [1.07-1.34]*
Fatty liver	any age	77,435	39,534,128	2.2	1.8	0.40	0.27	1.23 [1.20-1.25]*	1.14 [1.11-1.16]*
Oesophageal varices	18+	4,805	36,093,029	0.2	0.1	0.02	0.02	1.22 [1.11-1.33]*	1.12 [1.00-1.25]
Cholecystitis	any age	36,888	39,695,778	0.9	0.9	−0.02	0.04	1.11 [1.07-1.15]*	1.05 [1.01-1.08]*
**Metabolic**									
Metabolic syndrome	any age	1,592	40,196,325	0.0	0.0	0.01	0.01	1.27 [1.09-1.48]*	1.18 [1.00-1.40]
Obesity	any age	202,440	35,116,121	6.3	5.5	0.77	0.65	1.17 [1.15-1.19]*	1.12 [1.10-1.13]*
Diabetes mellitus	any age	211,379	33,877,918	6.4	6.2	0.24	0.40	1.10 [1.09-1.12]*	1.07 [1.05-1.08]*
Dyslipidaemia	any age	233,796	33,126,227	7.1	7.0	0.07	0.23	1.05 [1.04-1.07]*	1.03 [1.02-1.05]*
**Cardiovascular**									
Thromboembolic diseases	18+	99,377	34,536,743	3.1	2.8	0.35	0.48	1.23 [1.21-1.26]*	1.18 [1.15-1.20]*
Peripheral artery disease	18+	58,139	35,009,961	1.7	1.6	0.12	0.19	1.16 [1.13-1.19]*	1.12 [1.09-1.16]*
Heart failure	18+	178,114	34,500,526	5.1	5.2	−0.03	0.51	1.15 [1.13-1.17]*	1.11 [1.09-1.13]*

Outcome	Main cohort	events	person-years	Crude Rate (per 1,000 person-years) exposed	unexposed	Crude Rate difference	Estimated Rate difference[2]	Hazard ratio (99% confidence interval)[1] minimally-adjusted	comorbidity-adjusted
Myocardial infarction	18+	85,523	34,323,530	2.5	2.5	−0.05	0.12	1.08 [1.06-1.11]*	1.05 [1.03-1.07]*
Stroke	18+	161,564	34,482,030	4.5	4.8	−0.24	0.19	1.08 [1.06-1.10]*	1.04 [1.03-1.06]*
Coronary artery disease	18+	263,114	27,094,936	10.0	9.6	0.46	0.36	1.06 [1.04-1.07]*	1.04 [1.02-1.05]*
Hypertension	18+	310,729	20,645,566	16.3	14.5	1.82	0.32	1.03 [1.02-1.04]*	1.02 [1.01-1.03]*
**ADHD and autism**									
Autism	any age	20,061	40,063,554	0.7	0.4	0.26	0.09	1.19 [1.14-1.24]*	1.14 [1.09-1.20]*
ADHD	any age	13,611	40,092,048	0.4	0.3	0.14	0.01	1.11 [1.05-1.17]*	1.02 [0.97-1.09]
**Bone health**									
Spine fracture	18+	40,626	35,805,762	1.2	1.1	0.05	0.14	1.20 [1.16-1.24]*	1.13 [1.10-1.17]*
Osteoporosis	18+	146,892	33,853,196	4.5	4.3	0.16	0.47	1.16 [1.15-1.18]*	1.12 [1.10-1.14]*
Pelvis fracture	18+	24,446	35,932,429	0.7	0.7	−0.04	0.05	1.12 [1.07-1.17]*	1.08 [1.03-1.13]*
Hip fracture	18+	74,166	35,626,857	1.9	2.1	−0.20	0.13	1.08 [1.05-1.10]*	1.07 [1.04-1.10]*
Wrist fracture	18+	88,266	34,176,561	2.7	2.6	0.11	0.10	1.09 [1.07-1.12]*	1.04 [1.02-1.06]*
**Neurological**									
Peripheral neuropathies	18+	362,151	27,666,633	14.5	12.5	1.96	1.50	1.18 [1.17-1.19]*	1.12 [1.10-1.13]*
Epilepsy	any age	28,065	39,240,731	0.8	0.7	0.08	0.07	1.13 [1.09-1.18]*	1.10 [1.06-1.15]*
Migraine	any age	155,745	35,787,986	4.9	4.1	0.83	0.28	1.13 [1.11-1.15]*	1.06 [1.04-1.08]*
Vascular dementia	40+	41,612	26,075,152	1.5	1.6	−0.18	0.04	1.05 [1.02-1.09]*	1.03 [0.99-1.07]
Alzheimer's dementia	40+	68,619	25,894,807	2.4	2.7	−0.31	0.01	1.02 [0.99-1.05]	1.00 [0.98-1.03]
Multiple sclerosis	18+	4,581	35,967,823	0.1	0.1	0.01	0.00	1.03 [0.94-1.13]	0.99 [0.90-1.10]
Parkinson's disease	40+	25,377	26,007,909	0.9	1.0	−0.11	−0.03	0.99 [0.95-1.03]	0.97 [0.93-1.02]
**Mental health and substance use**									
Alcohol abuse	18+	32,986	35,210,385	1.1	0.9	0.21	0.10	1.16 [1.13-1.20]*	1.10 [1.06-1.15]*
Depression	any age	423,775	26,381,735	17.5	15.4	2.15	1.52	1.15 [1.14-1.16]*	1.09 [1.08-1.11]*
Anxiety	any age	436,589	30,278,081	16.1	13.7	2.39	1.30	1.16 [1.15-1.17]*	1.09 [1.08-1.10]*
Cigarette smoking	18+	389,069	11,869,938	34.7	31.8	2.87	0.89	1.06 [1.05-1.08]*	1.03 [1.02-1.04]*

[1] Hazard ratios (99% confidence intervals) estimated from Cox models comparing people with eczema to those without eczema. * indicates that the result is significant under Bonferroni-correction.

[2] Rate difference calculated using the estimated rate in the unexposed (rate in the exposed * (1/hazard ratio))

Supplementary Table 3: Eczema compared to no eczema (different cohorts)

	any age			18+			40+			<18			more severe eczema		
Outcome	events	person-years	HR (99% CI)	events	person-years	HR (99% CI)	events	person-years	HR (99% CI)	events	person-years	HR (99% CI)	events	person-years	HR (99% CI)
**Atopic and allergic**															
Food allergy	94,867	129,677,918	4.03 [3.95-4.11]*	36,346	90,758,416	2.00 [1.94-2.07]*	19,014	58,847,144	1.64 [1.56-1.72]*	67,435	52,722,951	5.30 [5.18-5.43]*	48,020	69,810,727	4.09 [3.99-4.21]*
Allergic Conjunctivitis	154,539	128,319,292	2.01 [1.98-2.04]*	83,746	88,908,159	2.05 [2.00-2.09]*	54,873	57,328,621	1.94 [1.89-1.99]*	79,747	52,924,451	1.95 [1.91-1.99]*	80,154	68,834,678	2.06 [2.01-2.10]*
Allergic Rhinitis	699,425	109,630,416	1.92 [1.90-1.93]*	346,822	72,658,422	1.64 [1.63-1.66]*	194,734	49,076,389	1.57 [1.55-1.59]*	401,482	46,324,198	2.11 [2.09-2.13]*	344,294	57,091,888	1.85 [1.83-1.87]*
Asthma	699,010	99,106,832	1.87 [1.86-1.89]*	342,326	65,228,770	1.56 [1.54-1.58]*	229,656	43,928,284	1.44 [1.42-1.46]*	391,020	42,163,744	2.11 [2.09-2.13]*	328,142	50,945,959	1.84 [1.82-1.86]*
Eosinophilic Oesophagitis	2,208	132,363,302	1.64 [1.42-1.90]*	1,830	92,362,564	1.39 [1.18-1.63]*	1,026	59,455,212	1.45 [1.18-1.79]*	850	54,412,995	1.73 [1.36-2.20]*	1,389	71,581,409	1.61 [1.33-1.93]*
**Cancer**															
Hodgkin lymphoma	3,522	132,300,421	1.85 [1.66-2.06]*	3,279	92,298,081	1.82 [1.63-2.04]*	2,123	59,406,647	1.83 [1.59-2.11]*	843	54,409,314	1.32 [1.04-1.69]	2,355	71,538,280	2.07 [1.81-2.35]*
Non-Hodgkin lymphoma	25,915	132,075,634	1.26 [1.21-1.32]*	25,409	92,075,310	1.25 [1.20-1.31]*	24,153	59,176,710	1.21 [1.16-1.27]*	958	54,407,241	1.44 [1.15-1.79]*	18,378	71,370,652	1.25 [1.19-1.32]*
Nonmelanoma skin cancer	267,492	128,656,533	1.13 [1.11-1.14]*	267,284	88,656,272	1.13 [1.12-1.15]*	263,245	55,764,474	1.13 [1.11-1.14]*	954	54,410,574	1.23 [0.97-1.57]	188,427	68,834,084	1.13 [1.11-1.15]*
Myeloma	10,458	132,301,340	1.10 [1.03-1.18]*	10,481	92,299,141	1.09 [1.02-1.17]	10,424	59,387,643	1.09 [1.01-1.16]	19	54,416,946	3.21 [0.30-34.91]	7,493	71,536,540	1.09 [1.00-1.18]
Melanoma	55,580	131,347,273	1.08 [1.05-1.11]*	54,902	91,358,254	1.06 [1.03-1.09]*	51,124	58,500,027	1.06 [1.03-1.09]*	1,717	54,383,646	1.36 [1.16-1.61]*	37,978	70,863,352	1.04 [1.00-1.08]
Pancreatic cancer	16,253	132,341,977	1.08 [1.02-1.14]	16,298	92,340,078	1.05 [1.00-1.11]	16,139	59,428,684	1.06 [1.00-1.12]	34	54,417,096	0.75 [0.16-3.61]	11,634	71,567,021	1.07 [1.00-1.14]
CNS cancers	27,380	132,126,472	1.05 [1.01-1.10]	26,029	92,134,082	1.04 [0.99-1.08]	23,961	59,252,865	1.04 [0.99-1.09]	2,149	54,393,808	1.07 [0.92-1.25]	18,383	71,426,502	1.01 [0.96-1.07]
Lung cancer	73,148	132,156,528	1.05 [1.02-1.08]*	73,294	92,155,590	1.05 [1.02-1.08]*	73,050	59,245,622	1.04 [1.01-1.07]*	133	54,415,892	0.81 [0.39-1.68]	51,939	71,441,203	1.04 [1.01-1.08]
Breast cancer	104,091	130,554,198	1.03 [1.01-1.06]*	103,962	90,553,313	1.03 [1.01-1.05]*	99,754	57,661,140	1.03 [1.00-1.05]	822	54,410,096	1.28 [0.99-1.65]	69,173	70,329,303	1.02 [0.99-1.04]
Prostate cancer	106,207	131,231,073	1.01 [0.99-1.03]	106,056	91,228,456	1.01 [0.99-1.03]	101,527	58,336,375	1.01 [0.99-1.03]	1,460	54,410,617	0.92 [0.74-1.14]	76,197	70,731,756	0.99 [0.96-1.01]
**Skin infection**															
Molluscum contagiosum	306,757	126,603,018	1.81 [1.79-1.83]*	12,208	89,759,059	1.58 [1.49-1.67]*	4,075	59,258,225	1.65 [1.50-1.82]*	297,997	49,124,962	1.82 [1.80-1.84]*	89,964	68,613,841	1.70 [1.67-1.74]*
Dermatophyte infection	774,374	112,045,992	1.60 [1.59-1.61]*	539,905	74,498,937	1.64 [1.63-1.66]*	368,210	46,928,806	1.65 [1.63-1.67]*	293,769	49,082,666	1.45 [1.43-1.46]*	422,121	58,121,050	1.58 [1.56-1.59]*
Herpes simplex	240,103	126,651,269	1.51 [1.49-1.53]*	170,625	87,318,770	1.49 [1.47-1.52]*	84,767	56,444,990	1.49 [1.46-1.52]*	103,444	52,701,653	1.48 [1.46-1.51]*	128,100	67,863,553	1.57 [1.54-1.60]*
Impetigo	513,128	118,822,044	1.49 [1.48-1.51]*	119,047	84,238,356	1.67 [1.64-1.70]*	59,605	56,884,350	1.70 [1.66-1.75]*	416,143	44,929,007	1.44 [1.43-1.45]*	173,066	64,011,567	1.64 [1.62-1.67]*
Cutaneous warts	1,102,269	109,295,803	1.30 [1.29-1.31]*	492,556	73,810,304	1.30 [1.29-1.32]*	356,688	49,250,317	1.27 [1.26-1.29]*	652,761	44,211,946	1.29 [1.28-1.30]*	509,152	57,467,979	1.24 [1.22-1.25]*
**Immune mediated**															
Alopecia Areata	34,376	131,681,350	1.77 [1.71-1.83]*	24,631	91,729,784	1.76 [1.69-1.83]*	10,296	59,101,543	1.87 [1.76-1.99]*	16,151	54,222,224	1.61 [1.53-1.69]*	19,376	71,069,501	2.12 [2.03-2.22]*
Urticaria	414,078	122,363,873	1.58 [1.57-1.60]*	219,498	84,661,641	1.77 [1.75-1.80]*	134,643	54,694,880	1.85 [1.82-1.88]*	226,520	50,282,124	1.38 [1.36-1.40]*	194,711	65,283,293	1.71 [1.69-1.74]*
COPD	283,421	127,542,421	1.22 [1.21-1.24]*	278,628	87,554,259	1.22 [1.21-1.24]*	269,930	54,706,063	1.22 [1.20-1.23]*	7,511	54,362,064	1.41 [1.30-1.52]*	192,615	68,086,813	1.23 [1.21-1.25]*
**Digestive system**															
Crohn's disease	20,230	131,801,877	1.62 [1.54-1.69]*	17,599	91,798,601	1.54 [1.46-1.61]*	10,036	59,063,492	1.41 [1.32-1.50]*	6,288	54,351,647	1.82 [1.67-1.97]*	12,678	71,135,928	1.64 [1.55-1.74]*
Coeliac disease	31,782	131,778,936	1.42 [1.37-1.47]*	22,873	91,838,275	1.49 [1.42-1.55]*	14,899	59,062,544	1.45 [1.38-1.53]*	12,507	54,283,960	1.34 [1.26-1.42]*	17,831	71,194,946	1.47 [1.40-1.55]*
Ulcerative colitis	27,027	131,570,373	1.40 [1.34-1.46]*	25,384	91,569,305	1.40 [1.35-1.46]*	16,743	58,799,312	1.37 [1.31-1.45]*	5,286	54,370,374	1.35 [1.23-1.49]*	17,388	70,989,662	1.41 [1.34-1.48]*
Irritable bowel syndrome	272,010	123,906,183	1.31 [1.29-1.32]*	251,882	83,877,969	1.30 [1.29-1.32]*	120,392	53,305,886	1.30 [1.27-1.32]*	76,481	53,686,842	1.22 [1.19-1.25]*	163,263	65,919,762	1.26 [1.24-1.28]*
Oesophagitis	248,927	125,676,829	1.25 [1.23-1.27]*	242,728	86,032,951	1.25 [1.23-1.26]*	205,543	53,626,422	1.23 [1.21-1.25]*	17,909	53,873,477	1.22 [1.16-1.28]*	164,123	66,957,966	1.24 [1.22-1.26]*
Gastro oesophageal reflux	539,815	120,056,979	1.25 [1.24-1.26]*	509,256	82,456,955	1.24 [1.23-1.26]*	374,993	50,974,376	1.24 [1.22-1.25]*	82,268	51,419,346	1.16 [1.13-1.19]*	343,899	63,928,212	1.23 [1.21-1.24]*
Gastritis and duodenitis	356,381	126,112,608	1.21 [1.20-1.23]*	324,992	86,343,605	1.21 [1.19-1.22]*	247,899	54,578,712	1.21 [1.19-1.23]*	62,694	53,672,691	1.16 [1.13-1.19]*	226,456	67,279,508	1.21 [1.19-1.22]*
Barrett's oesophagus	42,774	131,755,330	1.17 [1.13-1.21]*	42,753	91,753,003	1.16 [1.12-1.20]*	41,353	58,848,546	1.16 [1.12-1.20]*	457	54,414,691	1.35 [0.95-1.92]	30,211	71,129,424	1.18 [1.13-1.22]*

	any age			18+			40+			<18			more severe eczema		
Outcome	events	person-years	HR (99% CI)	events	person-years	HR (99% CI)	events	person-years	HR (99% CI)	events	person-years	HR (99% CI)	events	person-years	HR (99% CI)
Diverticular disease	314,315	127,736,124	1.17 [1.16-1.18]*	314,741	87,721,438	1.17 [1.15-1.18]*	310,590	54,835,505	1.17 [1.15-1.18]*	1,035	54,411,740	0.94 [0.73-1.21]	218,309	68,165,812	1.17 [1.15-1.18]*
Peptic ulcer disease	71,432	130,748,986	1.16 [1.13-1.19]*	70,949	90,751,917	1.15 [1.12-1.18]*	65,423	57,920,062	1.15 [1.12-1.18]*	2,186	54,395,190	1.09 [0.93-1.28]	48,774	70,429,325	1.16 [1.13-1.20]*
Abdominal hernia	291,201	125,008,052	1.13 [1.12-1.15]*	264,596	86,491,371	1.14 [1.13-1.16]*	231,055	54,288,859	1.13 [1.11-1.15]*	35,858	52,524,243	1.05 [1.01-1.09]*	186,986	66,947,242	1.13 [1.11-1.15]*
Pancreatitis	37,452	131,799,502	1.11 [1.07-1.15]*	36,836	91,796,093	1.11 [1.07-1.15]*	30,683	58,949,815	1.11 [1.06-1.15]*	2,838	54,396,908	1.05 [0.91-1.20]	25,305	71,181,445	1.13 [1.08-1.18]*
Appendicitis	109,685	129,865,984	1.07 [1.05-1.09]*	71,290	89,995,036	1.06 [1.03-1.09]*	31,752	58,320,822	1.06 [1.02-1.10]*	59,026	53,680,531	1.07 [1.04-1.10]*	58,000	70,025,325	1.04 [1.01-1.07]*
Peritonitis	13,032	132,170,298	1.07 [1.00-1.13]	11,937	92,175,888	1.09 [1.02-1.17]*	10,552	59,293,913	1.07 [1.00-1.15]	1,572	54,396,733	0.92 [0.77-1.10]	8,610	71,446,300	1.12 [1.04-1.21]*
**Liver**															
Autoimmune liver disease	6,352	132,269,823	1.32 [1.21-1.43]*	6,113	92,269,013	1.33 [1.22-1.45]*	5,445	59,366,418	1.28 [1.17-1.41]*	536	54,412,135	1.35 [0.99-1.84]	4,332	71,506,370	1.40 [1.27-1.56]*
Fibrosis/sclerosis/cirrhosis	29,310	132,025,070	1.27 [1.22-1.33]*	29,160	92,025,270	1.26 [1.21-1.32]*	27,441	59,122,489	1.26 [1.20-1.32]*	459	54,413,748	1.07 [0.73-1.57]	20,401	71,339,042	1.33 [1.27-1.40]*
Oesophageal varices	8,969	132,294,769	1.25 [1.15-1.36]*	8,813	92,294,383	1.19 [1.10-1.30]*	8,337	59,385,120	1.20 [1.10-1.30]*	222	54,415,204	0.68 [0.37-1.26]	6,264	71,530,708	1.31 [1.19-1.44]*
Fatty liver	148,815	131,108,036	1.24 [1.22-1.26]*	147,992	91,104,366	1.24 [1.22-1.26]*	125,327	58,292,663	1.22 [1.20-1.24]*	9,174	54,378,761	1.12 [1.04-1.21]*	100,970	70,678,948	1.27 [1.25-1.30]*
Cholecystitis	66,438	131,413,446	1.10 [1.07-1.14]*	66,188	91,410,660	1.10 [1.07-1.13]*	54,914	58,589,876	1.10 [1.06-1.13]*	3,750	54,393,261	1.06 [0.94-1.20]	45,105	70,908,953	1.09 [1.06-1.13]*
**Cardiovascular**															
Thromboembolic diseases	179,703	129,589,832	1.25 [1.23-1.27]*	177,337	89,591,235	1.25 [1.23-1.27]*	153,374	56,887,914	1.26 [1.24-1.28]*	10,466	54,349,762	1.08 [1.01-1.16]	121,924	69,538,408	1.29 [1.26-1.31]*
Peripheral artery disease	109,045	130,499,739	1.19 [1.17-1.22]*	103,675	90,508,776	1.18 [1.16-1.21]*	90,469	57,774,816	1.19 [1.16-1.22]*	11,096	54,318,982	1.25 [1.17-1.33]*	72,271	70,250,314	1.19 [1.16-1.22]*
Heart failure	271,334	129,977,066	1.16 [1.15-1.18]*	270,404	89,987,240	1.16 [1.15-1.18]*	267,064	57,096,857	1.16 [1.15-1.18]*	1,874	54,393,484	1.22 [1.03-1.43]	195,647	69,812,797	1.19 [1.17-1.21]*
Coronary artery disease	542,526	117,637,329	1.15 [1.14-1.16]*	540,912	77,641,301	1.15 [1.14-1.16]*	521,594	44,792,835	1.14 [1.13-1.15]*	2,550	54,392,243	1.16 [1.01-1.33]	355,004	61,020,143	1.14 [1.12-1.15]*
Hypertension	778,252	107,536,239	1.11 [1.10-1.12]*	775,356	67,565,647	1.11 [1.10-1.12]*	726,959	34,956,886	1.10 [1.09-1.10]*	12,608	54,314,974	1.15 [1.08-1.23]*	512,582	54,045,752	1.12 [1.11-1.13]*
Stroke	262,348	129,923,172	1.09 [1.08-1.11]*	261,037	89,936,081	1.09 [1.07-1.10]*	253,317	57,072,340	1.09 [1.07-1.10]*	4,262	54,383,050	1.09 [0.97-1.22]	185,120	69,782,157	1.10 [1.08-1.12]*
Myocardial infarction	151,921	129,599,396	1.09 [1.07-1.11]*	151,717	89,598,403	1.09 [1.07-1.11]*	149,802	56,718,012	1.08 [1.06-1.10]*	529	54,407,411	1.21 [0.87-1.67]	106,145	69,576,394	1.08 [1.06-1.11]*
**Metabolic**															
Metabolic syndrome	3,040	132,305,044	1.25 [1.10-1.42]*	2,914	92,303,697	1.25 [1.09-1.42]*	2,618	59,394,105	1.20 [1.05-1.37]*	225	54,415,577	1.34 [0.81-2.24]	1,978	71,540,170	1.21 [1.03-1.42]
Obesity	441,442	122,842,347	1.22 [1.21-1.23]*	413,075	82,975,060	1.21 [1.19-1.22]*	315,700	51,643,300	1.18 [1.17-1.20]*	60,129	53,677,040	1.24 [1.20-1.27]*	279,052	65,021,429	1.21 [1.20-1.23]*
Dyslipidaemia	472,695	120,777,185	1.13 [1.12-1.14]*	471,352	80,794,082	1.13 [1.11-1.14]*	443,724	48,106,476	1.11 [1.10-1.13]*	8,055	54,340,806	1.15 [1.06-1.25]*	312,780	63,323,703	1.12 [1.10-1.13]*
Diabetes mellitus	424,701	122,434,156	1.13 [1.12-1.14]*	412,898	82,513,267	1.13 [1.12-1.14]*	382,135	50,014,610	1.12 [1.11-1.13]*	21,153	54,175,049	1.03 [0.98-1.08]	284,447	64,552,155	1.14 [1.12-1.15]*
**Neurological**															
Peripheral neuropathies	749,054	115,839,998	1.21 [1.20-1.22]*	735,314	75,941,901	1.21 [1.20-1.22]*	572,444	44,549,919	1.20 [1.19-1.21]*	58,571	53,882,012	1.08 [1.05-1.11]*	485,557	60,000,225	1.21 [1.19-1.22]*
Migraine	433,361	122,281,709	1.18 [1.17-1.19]*	340,641	82,416,524	1.16 [1.15-1.18]*	142,854	53,446,927	1.16 [1.14-1.18]*	180,757	52,498,784	1.15 [1.13-1.17]*	244,301	65,057,469	1.15 [1.13-1.16]*
Epilepsy	79,458	129,897,303	1.15 [1.12-1.18]*	54,425	90,146,585	1.20 [1.16-1.23]*	36,696	57,939,223	1.22 [1.18-1.26]*	33,100	53,835,759	1.04 [1.01-1.08]	42,548	70,038,866	1.15 [1.11-1.19]*
Multiple sclerosis	10,738	131,992,849	1.08 [1.01-1.16]	10,608	91,991,931	1.11 [1.03-1.18]*	7,058	59,118,667	1.06 [0.97-1.15]	1,119	54,410,184	1.20 [0.96-1.50]	6,794	71,338,756	1.04 [0.96-1.14]
Vascular dementia	57,197	132,088,130	1.07 [1.04-1.11]*	57,484	92,084,536	1.05 [1.02-1.09]*	57,217	59,173,934	1.07 [1.04-1.10]*	2	54,417,313	45.45 [0.00-Inf]	42,203	71,382,082	1.06 [1.02-1.09]*
Alzheimer's dementia	101,292	131,809,395	1.04 [1.01-1.06]*	101,212	91,807,655	1.03 [1.01-1.06]*	101,271	58,896,196	1.04 [1.02-1.06]*	7	54,417,299	0.00 [0.00-Inf]	75,030	71,183,422	1.04 [1.01-1.07]*
Parkinson's disease	41,294	131,920,159	1.01 [0.98-1.05]	41,246	91,918,870	1.01 [0.98-1.05]	40,973	59,007,426	1.02 [0.98-1.05]	42	54,417,083	1.68 [0.28-10.02]	29,252	71,271,015	1.00 [0.96-1.04]
**Bone health**															
Osteoporosis	250,761	128,703,828	1.18 [1.17-1.20]*	249,956	88,699,650	1.18 [1.16-1.20]*	239,610	55,888,565	1.18 [1.16-1.20]*	6,147	54,374,325	1.03 [0.94-1.14]	171,836	68,929,999	1.19 [1.17-1.21]*
Spine fracture	69,735	131,767,311	1.18 [1.15-1.21]*	67,844	91,764,889	1.17 [1.14-1.20]*	62,684	58,938,370	1.18 [1.15-1.21]*	4,456	54,380,303	1.08 [0.97-1.20]	49,456	71,150,690	1.19 [1.15-1.23]*
Pelvis fracture	40,804	132,000,002	1.11 [1.07-1.16]*	39,320	91,998,149	1.11 [1.07-1.15]*	36,635	59,146,906	1.10 [1.06-1.15]*	2,892	54,389,448	1.13 [0.99-1.29]	28,790	71,326,578	1.15 [1.10-1.20]*
Hip fracture	115,400	131,556,904	1.10 [1.08-1.13]*	114,345	91,558,912	1.11 [1.09-1.13]*	112,923	58,674,498	1.11 [1.09-1.13]*	1,675	54,397,140	1.01 [0.85-1.20]	82,664	70,997,170	1.12 [1.09-1.15]*

Outcome	any age			18+			40+			<18			more severe eczema		
	events	person-years	HR (99% CI)	events	person-years	HR (99% CI)	events	person-years	HR (99% CI)	events	person-years	HR (99% CI)	events	person-years	HR (99% CI)
Wrist fracture	347,340	126,339,972	1.07 [1.06-1.08]*	209,406	86,928,363	1.07 [1.05-1.08]*	141,676	56,549,751	1.08 [1.06-1.10]*	176,301	52,360,923	1.05 [1.04-1.07]*	182,701	67,833,624	1.06 [1.04-1.08]*
**Mental health and substance use**															
Depression	1,173,853	101,880,802	1.17 [1.16-1.17]*	1,057,782	61,734,001	1.18 [1.17-1.18]*	503,245	37,862,921	1.19 [1.18-1.20]*	412,429	51,317,877	1.07 [1.06-1.08]*	694,183	51,515,079	1.17 [1.16-1.18]*
Anxiety	1,192,074	110,373,722	1.17 [1.16-1.17]*	1,036,290	70,517,871	1.16 [1.15-1.17]*	500,440	44,263,250	1.17 [1.16-1.18]*	442,174	51,489,247	1.12 [1.11-1.13]*	703,338	57,090,892	1.14 [1.14-1.15]*
Cigarette smoking	1,503,334	75,626,953	1.16 [1.15-1.16]*	1,152,377	36,461,715	1.11 [1.11-1.12]*	676,513	21,652,862	1.11 [1.10-1.12]*	597,160	46,705,054	1.20 [1.19-1.22]*	842,247	34,466,678	1.16 [1.15-1.17]*
Alcohol abuse	86,553	130,161,057	1.14 [1.11-1.16]*	85,997	90,151,061	1.13 [1.10-1.15]*	59,149	57,593,204	1.17 [1.14-1.21]*	9,263	54,340,238	1.00 [0.92-1.08]	56,007	70,101,808	1.16 [1.13-1.20]*
**ADHD and autism**															
Autism	117,398	131,252,965	1.15 [1.13-1.17]*	20,490	91,818,977	1.18 [1.12-1.24]*	5,087	59,391,025	1.59 [1.45-1.76]*	108,483	53,464,948	1.12 [1.10-1.15]*	43,049	71,036,201	1.12 [1.09-1.16]*
ADHD	82,899	131,446,061	1.08 [1.06-1.11]*	17,820	91,837,352	1.04 [0.98-1.10]	3,667	59,423,507	1.05 [0.93-1.19]	75,154	53,631,908	1.07 [1.05-1.10]*	32,206	71,140,671	1.05 [1.01-1.09]

Hazard ratios (99% confidence intervals) estimated from Cox models comparing people with eczema to those without eczema. * indicates that the result is significant under Bonferroni-correction.

Supplementary Table 4: Mild, moderate, and severe eczema compared to no eczema

													Hazard ratio (99% confidence interval)[1]					
	Event				Person-years				Rate (per 1,000 person-years)				comorbidity-adjusted			+ hospital-admission-adjusted		
Outcome	unexp.	mild	mod	sev.	unexposed	mild	moderate	severe	unexp.	mild	mod.	sev.	mild	moderate	severe	mild	moderate	severe
**Atopic and allergic**																		
Asthma	387,024	121,967	71,947	2,087	64,159,288	9,827,726	6,899,893	182,998	6.0	12.4	10.4	11.4	1.82 [1.81-1.84]*	1.93 [1.91-1.95]*	2.27 [2.12-2.42]*	1.75 [1.74-1.77]*	1.84 [1.82-1.86]*	1.87 [1.75-2.00]*
Food allergy	37,366	26,076	15,433	756	84,873,276	12,122,364	9,013,238	258,269	0.4	2.2	1.7	2.9	3.80 [3.72-3.89]*	4.41 [4.28-4.55]*	7.19 [6.03-8.57]*	3.66 [3.59-3.75]*	4.19 [4.06-4.32]*	5.72 [4.81-6.80]*
Allergic Rhinitis	380,456	125,412	77,327	2,049	71,568,278	10,480,431	7,501,680	215,419	5.3	12.0	10.3	9.5	1.85 [1.84-1.87]*	1.98 [1.96-2.00]*	2.10 [1.96-2.24]*	1.82 [1.80-1.83]*	1.94 [1.92-1.96]*	1.93 [1.80-2.07]*
Allergic Conjunctivitis	82,808	26,404	19,795	622	83,945,264	12,073,779	8,870,245	258,534	1.0	2.2	2.2	2.4	1.83 [1.80-1.87]*	2.22 [2.17-2.26]*	2.62 [2.30-2.98]*	1.80 [1.77-1.83]*	2.16 [2.12-2.21]*	2.43 [2.14-2.77]*
Eosinophilic Oesophagitis	1,202	315	263	18	86,543,079	12,436,221	9,236,836	272,835	0.0	0.0	0.0	0.1	1.64 [1.37-1.96]*	1.64 [1.33-2.02]*	2.94 [1.22-7.07]	1.50 [1.23-1.83]*	1.27 [1.00-1.61]	1.86 [0.77-4.47]
**Immune mediated**																		
Alopecia Areata	19,433	4,498	4,703	146	86,089,509	12,394,067	9,168,935	270,395	0.2	0.4	0.5	0.5	1.47 [1.41-1.53]*	2.20 [2.11-2.30]*	2.31 [1.79-2.99]*	1.45 [1.38-1.51]*	2.16 [2.06-2.26]*	2.18 [1.69-2.81]*
Urticaria	241,612	60,901	41,024	1,062	79,965,865	11,568,010	8,477,508	244,685	3.0	5.3	4.8	4.3	1.43 [1.41-1.44]*	1.83 [1.80-1.86]*	1.76 [1.61-1.93]*	1.40 [1.38-1.41]*	1.78 [1.75-1.80]*	1.61 [1.47-1.76]*
COPD	169,013	20,065	32,716	1,427	36,380,998	3,626,581	4,582,128	158,940	4.6	5.5	7.1	9.0	1.16 [1.14-1.18]*	1.22 [1.20-1.24]*	1.61 [1.48-1.75]*	1.11 [1.08-1.13]*	1.16 [1.14-1.18]*	1.45 [1.33-1.58]*
**Mental health and substance use**																		
Anxiety	736,069	131,842	114,622	3,515	71,543,556	11,113,645	7,707,059	219,381	10.3	11.9	14.9	16.0	1.15 [1.14-1.16]*	1.17 [1.16-1.18]*	1.15 [1.09-1.20]*	1.11 [1.11-1.12]*	1.13 [1.12-1.14]*	1.04 [0.99-1.09]
Depression	724,983	124,504	118,326	3,870	65,556,507	10,701,833	7,110,763	189,898	11.1	11.6	16.6	20.4	1.13 [1.12-1.14]*	1.18 [1.17-1.19]*	1.45 [1.39-1.53]*	1.08 [1.07-1.09]*	1.12 [1.11-1.13]*	1.25 [1.19-1.31]*
Alcohol abuse	55,308	6,977	8,859	242	59,686,892	6,542,066	7,183,824	231,181	0.9	1.1	1.2	1.0	1.07 [1.04-1.11]*	1.16 [1.12-1.19]*	1.02 [0.84-1.23]	1.02 [0.98-1.05]	1.08 [1.05-1.12]*	0.88 [0.73-1.07]
Cigarette smoking	711,589	120,980	121,543	3,509	22,481,669	3,361,470	3,345,392	96,503	31.7	36.0	36.3	36.4	1.07 [1.06-1.08]*	1.12 [1.11-1.13]*	1.16 [1.10-1.22]*	1.06 [1.05-1.07]*	1.10 [1.09-1.11]*	1.11 [1.05-1.16]*
**ADHD and autism**																		
ADHD	49,693	13,310	3,881	93	85,934,630	12,337,640	9,200,286	271,914	0.6	1.1	0.4	0.3	1.07 [1.04-1.09]*	1.10 [1.05-1.15]*	1.15 [0.85-1.57]	1.02 [0.99-1.05]	1.03 [0.98-1.08]	0.92 [0.67-1.24]
Autism	70,460	19,514	5,546	174	85,818,009	12,304,269	9,186,268	271,592	0.8	1.6	0.6	0.6	1.14 [1.11-1.16]*	1.19 [1.15-1.24]*	1.54 [1.22-1.93]*	1.09 [1.06-1.11]*	1.12 [1.08-1.17]*	1.19 [0.94-1.49]
**Cardiovascular**																		
Hypertension	485,707	58,437	91,668	3,494	43,923,375	5,509,536	5,337,945	158,419	11.1	10.6	17.2	22.1	1.06 [1.05-1.07]*	1.11 [1.10-1.12]*	1.35 [1.29-1.42]*	1.04 [1.02-1.05]*	1.08 [1.07-1.09]*	1.27 [1.21-1.34]*
Coronary artery disease	347,084	38,716	60,931	2,076	51,039,193	5,962,107	6,102,903	190,493	6.8	6.5	10.0	10.9	1.12 [1.11-1.14]*	1.13 [1.12-1.14]*	1.28 [1.20-1.37]*	1.07 [1.06-1.09]*	1.08 [1.06-1.09]*	1.14 [1.07-1.21]*
Peripheral artery disease	66,993	7,507	11,563	550	59,937,960	6,556,443	7,183,579	228,595	1.1	1.1	1.6	2.4	1.12 [1.08-1.15]*	1.18 [1.15-1.21]*	1.74 [1.53-1.98]*	1.06 [1.03-1.10]*	1.12 [1.09-1.15]*	1.54 [1.36-1.75]*
Myocardial infarction	99,087	9,339	16,701	726	59,315,205	6,535,076	7,122,584	227,836	1.7	1.4	2.3	3.2	1.03 [1.00-1.06]	1.09 [1.07-1.11]*	1.54 [1.38-1.72]*	0.93 [0.90-0.96]*	0.98 [0.96-1.00]	1.27 [1.14-1.41]*
Stroke	170,968	16,663	28,922	1,159	59,571,573	6,529,608	7,118,675	227,756	2.9	2.6	4.1	5.1	1.05 [1.03-1.07]*	1.09 [1.07-1.10]*	1.37 [1.26-1.49]*	0.97 [0.95-0.99]*	1.00 [0.99-1.02]	1.18 [1.09-1.29]*
Heart failure	174,440	17,113	32,869	1,500	59,596,819	6,540,107	7,125,567	226,876	2.9	2.6	4.6	6.6	1.08 [1.05-1.10]*	1.18 [1.16-1.20]*	1.81 [1.67-1.96]*	0.96 [0.94-0.98]*	1.06 [1.04-1.08]*	1.48 [1.36-1.60]*
Thromboembolic diseases	113,962	13,113	21,175	1,113	59,328,977	6,517,241	7,086,174	222,921	1.9	2.0	3.0	5.0	1.14 [1.12-1.17]*	1.28 [1.26-1.31]*	2.05 [1.87-2.24]*	1.04 [1.01-1.07]*	1.17 [1.15-1.19]*	1.67 [1.53-1.83]*
**Metabolic**																		
Obesity	279,334	39,284	48,973	1,562	79,960,812	11,906,478	8,481,447	243,648	3.5	3.3	5.8	6.4	1.17 [1.16-1.19]*	1.23 [1.21-1.24]*	1.28 [1.19-1.37]*	1.14 [1.13-1.16]*	1.19 [1.17-1.20]*	1.19 [1.10-1.28]*
Dyslipidaemia	302,811	35,088	51,990	1,768	78,341,658	11,899,071	8,334,504	239,685	3.9	2.9	6.2	7.4	1.12 [1.10-1.13]*	1.11 [1.10-1.13]*	1.13 [1.06-1.21]*	1.09 [1.08-1.11]*	1.08 [1.07-1.10]*	1.07 [1.00-1.14]
Diabetes mellitus	271,217	30,899	48,577	1,897	79,520,034	12,002,507	8,509,284	243,340	3.4	2.6	5.7	7.8	1.07 [1.05-1.08]*	1.15 [1.14-1.17]*	1.29 [1.21-1.38]*	1.03 [1.01-1.05]*	1.11 [1.09-1.12]*	1.18 [1.11-1.27]*
Metabolic syndrome	1,938	253	355	17	86,499,544	12,433,841	9,232,435	272,667	0.0	0.0	0.0	0.1	1.30 [1.08-1.57]*	1.18 [1.01-1.38]	1.61 [0.81-3.19]	1.22 [1.01-1.47]	1.07 [0.91-1.25]	1.41 [0.72-2.79]
**Bone health**																		
Hip fracture	75,355	7,124	12,525	546	60,693,513	6,603,614	7,258,978	232,448	1.2	1.1	1.7	2.3	1.05 [1.02-1.09]*	1.10 [1.07-1.13]*	1.89 [1.66-2.14]*	0.95 [0.92-0.98]*	1.00 [0.97-1.02]	1.63 [1.43-1.85]*
Pelvis fracture	25,728	2,477	4,279	238	60,999,869	6,623,580	7,293,289	233,997	0.4	0.4	0.6	1.0	1.04 [0.98-1.10]	1.10 [1.06-1.15]*	1.98 [1.62-2.42]*	0.94 [0.89-1.00]	1.01 [0.96-1.05]	1.67 [1.36-2.04]*
Spine fracture	43,446	4,514	7,904	499	60,842,269	6,612,301	7,272,339	232,672	0.7	0.7	1.1	2.1	1.10 [1.06-1.15]*	1.18 [1.14-1.22]*	2.12 [1.84-2.44]*	1.00 [0.96-1.04]	1.08 [1.05-1.12]*	1.80 [1.56-2.08]*

													Hazard ratio (99% confidence interval)[1]					
	Event				Person-years				Rate (per 1,000 person-years)				comorbidity-adjusted			+ hospital-admission-adjusted		
Outcome	unexp.	mild	mod	sev.	unexposed	mild	moderate	severe	unexp.	mild	mod.	sev.	mild	moderate	severe	mild	moderate	severe
Wrist fracture	137,628	17,277	18,838	733	57,381,570	6,336,699	7,038,331	224,751	2.4	2.7	2.7	3.3	1.04 [1.01-1.06]*	1.08 [1.05-1.10]*	1.18 [1.07-1.31]*	1.01 [0.99-1.03]	1.03 [1.01-1.05]*	1.08 [0.98-1.20]
Osteoporosis	159,261	17,359	27,775	2,246	58,714,552	6,468,489	7,032,838	211,907	2.7	2.7	3.9	10.6	1.13 [1.10-1.15]*	1.14 [1.13-1.16]*	3.07 [2.86-3.29]*	1.05 [1.03-1.07]*	1.07 [1.05-1.09]*	2.70 [2.52-2.90]*
**Skin infection**																		
Molluscum contagiosum	170,337	69,859	16,131	359	82,977,380	11,573,925	8,916,324	265,431	2.1	6.0	1.8	1.4	1.76 [1.74-1.78]*	2.01 [1.97-2.06]*	2.19 [1.86-2.58]*	1.76 [1.74-1.78]*	2.01 [1.96-2.06]*	2.17 [1.84-2.55]*
Impetigo	303,121	88,959	33,445	876	77,774,077	10,971,946	8,389,802	248,230	3.9	8.1	4.0	3.5	1.39 [1.38-1.41]*	1.75 [1.72-1.78]*	2.09 [1.88-2.31]*	1.38 [1.36-1.39]*	1.72 [1.69-1.75]*	1.92 [1.73-2.13]*
Herpes simplex	141,257	31,698	25,822	1,176	82,752,931	11,997,878	8,799,724	252,449	1.7	2.6	2.9	4.7	1.40 [1.38-1.42]*	1.60 [1.58-1.63]*	2.74 [2.49-3.02]*	1.36 [1.34-1.39]*	1.55 [1.53-1.58]*	2.50 [2.27-2.75]*
Dermatophyte infection	462,027	102,824	85,289	2,170	73,015,310	10,874,722	7,575,301	223,471	6.3	9.5	11.3	9.7	1.51 [1.50-1.53]*	1.66 [1.65-1.68]*	1.51 [1.42-1.60]*	1.49 [1.47-1.50]*	1.62 [1.61-1.64]*	1.41 [1.33-1.50]*
Cutaneous warts	675,992	155,589	84,401	2,452	71,210,053	10,403,586	7,700,518	223,759	9.5	15.0	11.0	11.0	1.29 [1.28-1.30]*	1.28 [1.27-1.29]*	1.39 [1.31-1.47]*	1.27 [1.26-1.28]*	1.26 [1.25-1.28]*	1.33 [1.26-1.41]*
**Cancer**																		
Lung cancer	48,767	4,333	7,762	366	61,107,638	6,633,029	7,304,030	234,473	0.8	0.7	1.1	1.6	1.05 [1.00-1.09]	1.03 [1.00-1.07]	1.48 [1.26-1.73]*	0.88 [0.84-0.92]*	0.89 [0.86-0.92]*	1.14 [0.97-1.35]
Breast cancer	69,450	6,654	9,937	320	59,969,003	6,562,898	7,196,345	231,456	1.2	1.0	1.4	1.4	1.03 [1.00-1.06]	1.02 [0.99-1.05]	0.85 [0.74-0.99]	1.00 [0.96-1.03]	0.99 [0.96-1.01]	0.79 [0.69-0.92]*
Prostate cancer	67,668	5,723	10,249	288	38,876,209	3,800,308	4,880,263	173,530	1.7	1.5	2.1	1.7	1.02 [0.99-1.06]	1.00 [0.97-1.02]	0.83 [0.72-0.97]	0.93 [0.90-0.97]*	0.91 [0.89-0.94]*	0.71 [0.61-0.83]*
Pancreatic cancer	10,846	933	1,710	63	61,239,895	6,640,609	7,317,273	235,170	0.2	0.1	0.2	0.3	1.03 [0.94-1.13]	1.05 [0.98-1.12]	1.14 [0.80-1.62]	0.87 [0.79-0.95]*	0.91 [0.85-0.98]*	0.96 [0.67-1.38]
Non-Hodgkin lymphoma	16,834	1,692	3,031	247	86,338,584	12,424,494	9,214,794	271,288	0.2	0.1	0.3	0.9	1.18 [1.10-1.26]*	1.26 [1.19-1.32]*	2.99 [2.44-3.67]*	0.98 [0.92-1.05]	1.08 [1.02-1.14]*	2.40 [1.95-2.97]*
Hodgkin lymphoma	2,031	332	518	25	86,499,683	12,433,423	9,232,218	272,705	0.0	0.0	0.1	0.1	1.53 [1.31-1.78]*	2.05 [1.80-2.34]*	3.06 [1.62-5.77]*	1.13 [0.95-1.35]	1.58 [1.37-1.82]*	2.02 [1.03-3.98]
Myeloma	6,989	609	1,145	36	86,496,646	12,434,320	9,232,490	272,718	0.1	0.0	0.1	0.1	1.06 [0.95-1.18]	1.12 [1.03-1.22]*	0.92 [0.59-1.45]	0.93 [0.83-1.04]	0.98 [0.90-1.06]	0.70 [0.44-1.11]
CNS cancers	18,273	1,834	2,625	94	86,372,194	12,424,802	9,222,229	272,373	0.2	0.1	0.3	0.3	1.02 [0.96-1.09]	1.03 [0.98-1.09]	1.10 [0.83-1.45]	0.87 [0.81-0.93]*	0.89 [0.85-0.94]*	0.85 [0.64-1.13]
Melanoma	36,664	3,537	5,433	198	85,842,328	12,391,904	9,166,167	270,364	0.4	0.3	0.6	0.7	1.06 [1.02-1.11]*	1.05 [1.02-1.09]*	1.09 [0.90-1.33]	1.00 [0.95-1.04]	0.99 [0.95-1.03]	0.95 [0.78-1.16]
Nonmelanoma skin cancer	175,588	16,460	29,747	1,382	83,933,378	12,273,300	8,932,476	260,623	2.1	1.3	3.3	5.3	1.08 [1.05-1.10]*	1.12 [1.10-1.14]*	1.64 [1.52-1.77]*	1.02 [1.00-1.04]	1.06 [1.05-1.08]*	1.48 [1.37-1.60]*
**Neurological**																		
Alzheimer's dementia	67,260	5,852	10,908	340	39,266,331	3,817,460	4,925,947	174,731	1.7	1.5	2.2	1.9	1.03 [0.99-1.06]	1.03 [1.00-1.06]	1.10 [0.95-1.27]	1.00 [0.96-1.04]	1.00 [0.98-1.03]	1.05 [0.91-1.22]
Vascular dementia	37,659	3,478	6,304	174	39,465,238	3,831,130	4,945,359	175,369	1.0	0.9	1.3	1.0	1.05 [1.00-1.10]	1.07 [1.03-1.11]*	1.09 [0.88-1.35]	0.96 [0.92-1.01]	0.99 [0.95-1.03]	0.98 [0.79-1.22]
Epilepsy	50,917	8,715	6,574	229	84,799,914	12,294,877	9,094,294	267,726	0.6	0.7	0.7	0.9	1.12 [1.09-1.16]*	1.18 [1.14-1.22]*	1.48 [1.23-1.78]*	1.01 [0.97-1.04]	1.03 [0.99-1.07]	1.06 [0.87-1.28]
Migraine	269,624	50,317	39,268	1,103	79,588,116	11,804,793	8,563,456	249,441	3.4	4.3	4.6	4.4	1.16 [1.15-1.18]*	1.18 [1.16-1.19]*	1.15 [1.05-1.25]*	1.12 [1.11-1.14]*	1.13 [1.11-1.15]*	1.03 [0.95-1.13]
Multiple sclerosis	6,980	866	936	33	60,989,085	6,624,344	7,297,474	234,343	0.1	0.1	0.1	0.1	1.14 [1.03-1.25]*	1.04 [0.95-1.14]	0.92 [0.58-1.46]	1.04 [0.95-1.15]	0.94 [0.86-1.03]	0.71 [0.44-1.14]
Parkinson's disease	27,442	2,449	4,059	109	39,337,778	3,827,177	4,937,813	175,116	0.7	0.6	0.8	0.6	1.04 [0.99-1.10]	0.99 [0.94-1.03]	0.82 [0.63-1.05]	0.99 [0.94-1.05]	0.94 [0.90-0.98]*	0.75 [0.58-0.97]
Peripheral neuropathies	464,270	61,511	83,936	2,933	49,880,855	5,814,907	6,015,448	180,698	9.3	10.6	14.0	16.2	1.16 [1.15-1.18]*	1.21 [1.20-1.23]*	1.31 [1.25-1.39]*	1.12 [1.11-1.14]*	1.17 [1.16-1.18]*	1.20 [1.14-1.27]*
**Digestive system**																		
Abdominal hernia	188,874	22,751	30,204	1,058	81,367,055	11,998,005	8,776,837	256,044	2.3	1.9	3.4	4.1	1.10 [1.08-1.12]*	1.13 [1.11-1.14]*	1.25 [1.15-1.36]*	1.05 [1.03-1.07]*	1.07 [1.05-1.09]*	1.10 [1.01-1.20]
Appendicitis	70,087	12,418	8,107	236	84,806,448	12,285,394	9,102,410	267,906	0.8	1.0	0.9	0.9	1.07 [1.04-1.10]*	1.04 [1.01-1.08]*	1.13 [0.94-1.36]	0.93 [0.91-0.96]*	0.89 [0.86-0.92]*	0.74 [0.61-0.90]*
Barrett's oesophagus	27,807	2,749	5,021	193	60,825,650	6,615,304	7,267,974	232,861	0.5	0.4	0.7	0.8	1.09 [1.04-1.15]*	1.20 [1.15-1.25]*	1.46 [1.19-1.78]*	0.94 [0.89-0.99]	1.03 [0.99-1.07]	1.12 [0.91-1.38]
Coeliac disease	19,167	3,856	3,337	101	86,144,296	12,398,330	9,193,597	270,823	0.2	0.3	0.4	0.4	1.31 [1.25-1.37]*	1.55 [1.47-1.63]*	1.42 [1.07-1.90]	1.17 [1.11-1.23]*	1.36 [1.29-1.44]*	1.01 [0.74-1.38]
Crohn's disease	11,874	2,245	2,415	375	86,157,628	12,415,540	9,204,808	248,179	0.1	0.2	0.3	1.5	1.44 [1.35-1.53]*	1.57 [1.48-1.67]*	7.58 [5.85-9.82]*	1.14 [1.06-1.22]*	1.29 [1.20-1.38]*	4.92 [3.74-6.47]*
Diverticular disease	201,955	21,521	37,085	1,586	58,032,259	6,429,695	6,929,883	218,588	3.5	3.3	5.4	7.3	1.12 [1.10-1.14]*	1.18 [1.16-1.20]*	1.37 [1.28-1.48]*	1.01 [0.99-1.03]	1.07 [1.06-1.09]*	1.15 [1.07-1.24]*
Gastritis and duodenitis	225,572	32,461	38,784	1,728	82,242,104	12,080,262	8,761,565	250,541	2.7	2.7	4.4	6.9	1.17 [1.15-1.19]*	1.22 [1.20-1.24]*	1.71 [1.59-1.84]*	1.07 [1.05-1.08]*	1.11 [1.09-1.13]*	1.39 [1.29-1.49]*
Gastro oesophageal reflux	336,990	50,332	60,606	2,060	78,092,572	11,629,111	8,390,929	240,190	4.3	4.3	7.2	8.6	1.22 [1.20-1.23]*	1.26 [1.24-1.27]*	1.27 [1.19-1.35]*	1.15 [1.14-1.17]*	1.19 [1.17-1.20]*	1.11 [1.05-1.19]*
Irritable bowel syndrome	166,652	29,947	29,223	654	80,708,246	11,955,442	8,627,782	250,175	2.1	2.5	3.4	2.6	1.29 [1.27-1.31]*	1.31 [1.29-1.33]*	1.03 [0.92-1.14]	1.24 [1.22-1.26]*	1.25 [1.23-1.27]*	0.90 [0.81-1.01]
Oesophagitis	156,201	20,150	29,242	1,126	81,932,969	12,093,532	8,733,800	251,587	1.9	1.7	3.3	4.5	1.20 [1.18-1.23]*	1.25 [1.23-1.27]*	1.54 [1.42-1.68]*	1.11 [1.09-1.13]*	1.14 [1.12-1.16]*	1.26 [1.15-1.37]*

													Hazard ratio (99% confidence interval)[1]					
	Event				Person-years				Rate (per 1,000 person-years)				comorbidity-adjusted			+ hospital-admission-adjusted		
Outcome	unexp.	mild	mod	sev.	unexposed	mild	moderate	severe	unexp.	mild	mod.	sev.	mild	moderate	severe	mild	moderate	severe
Pancreatitis	24,188	2,674	4,038	208	86,146,621	12,412,032	9,196,622	270,591	0.3	0.2	0.4	0.8	1.05 [0.99-1.11]	1.12 [1.07-1.17]*	1.56 [1.26-1.93]*	0.89 [0.85-0.95]*	0.96 [0.91-1.00]	1.19 [0.96-1.49]
Peptic ulcer disease	46,429	4,841	8,064	390	85,406,187	12,369,807	9,123,197	267,096	0.5	0.4	0.9	1.5	1.09 [1.05-1.13]*	1.17 [1.14-1.21]*	1.77 [1.52-2.06]*	0.95 [0.91-0.99]*	1.03 [1.00-1.07]	1.36 [1.17-1.59]*
Peritonitis	8,556	922	1,270	115	86,405,485	12,427,935	9,225,173	271,377	0.1	0.1	0.1	0.4	1.02 [0.93-1.12]	1.03 [0.95-1.11]	3.04 [2.21-4.18]*	0.84 [0.77-0.93]*	0.88 [0.81-0.95]*	1.99 [1.44-2.76]*
Ulcerative colitis	16,573	2,464	3,021	310	85,987,330	12,404,905	9,186,120	254,361	0.2	0.2	0.3	1.2	1.30 [1.22-1.37]*	1.38 [1.31-1.46]*	3.76 [3.01-4.69]*	1.12 [1.06-1.19]*	1.19 [1.12-1.25]*	2.62 [2.08-3.31]*
**Liver**																		
Autoimmune liver disease	3,946	486	728	116	86,476,529	12,433,716	9,231,398	269,582	0.0	0.0	0.1	0.4	1.15 [1.01-1.31]	1.29 [1.16-1.43]*	6.93 [4.54-10.58]*	1.02 [0.89-1.16]	1.14 [1.02-1.27]	5.32 [3.46-8.18]*
Cholecystitis	43,144	4,728	7,070	309	85,879,139	12,392,048	9,166,046	269,714	0.5	0.4	0.8	1.1	1.07 [1.03-1.11]*	1.10 [1.06-1.14]*	1.36 [1.16-1.60]*	0.93 [0.89-0.97]*	0.96 [0.93-0.99]	1.09 [0.93-1.28]
Fatty liver	93,163	12,052	18,162	1,005	85,692,787	12,365,210	9,120,529	266,646	1.1	1.0	2.0	3.8	1.14 [1.11-1.17]*	1.29 [1.26-1.31]*	1.78 [1.61-1.96]*	1.07 [1.05-1.10]*	1.20 [1.18-1.23]*	1.54 [1.40-1.71]*
Fibrosis/sclerosis/cirrhosis	18,142	2,005	3,735	239	61,017,753	6,627,845	7,294,327	232,809	0.3	0.3	0.5	1.0	1.11 [1.03-1.19]*	1.33 [1.27-1.40]*	2.53 [2.01-3.20]*	0.95 [0.88-1.02]	1.16 [1.10-1.23]*	2.08 [1.64-2.64]*
Oesophageal varices	5,531	567	1,136	95	61,207,760	6,638,838	7,313,764	234,379	0.1	0.1	0.2	0.4	1.07 [0.93-1.23]	1.22 [1.11-1.35]*	3.05 [2.04-4.54]*	0.85 [0.73-0.99]	1.01 [0.91-1.12]	2.56 [1.67-3.92]*

[1] Hazard ratios (99% confidence intervals) estimated from Cox models comparing people with mild, moderate or severe eczema to those without eczema. * indicates that the result is significant under Bonferroni-correction.

Supplementary Table 5: Most commonly occurring codes

Outcome	Numbered list of the most commonly occurring codes with rounded number of occurrences and cumulative percentage of all occurrences
ADHD	1. Attention deficit hyperactivity disorder 639,300 (72.5%); 2. Attention deficit with hyperactivity 65,400 (79.9%); 3. Child attention deficit disorder 55,200 (86.1%); 4. Hyperactive behaviour 35,900 (90.2%); 5. Attention deficit disorder 33,000 (94.0%)
Abdominal hernia	1. Inguinal hernia 782,300 (22.1%); 2. Primary repair of inguinal hernia 557,200 (37.8%); 3. Umbilical hernia 484,300 (51.4%); 4. Right inguinal hernia 322,200 (60.5%); 5. Left inguinal hernia 246,000 (67.4%); 6. Repair of umbilical hernia 177,700 (72.4%); 7. Paraumbilical hernia 141,900 (76.4%); 8. Primary mesh repair of inguinal hernia 61,300 (78.2%); 9. Inguinal hernia NOS 46,700 (79.5%); 10. Ventral hernia 44,500 (80.7%); 11. Bilateral inguinal hernia repair 39,400 (81.8%); 12. Primary laparoscopic repair of inguinal hernia 36,200 (82.9%); 13. Femoral hernia 35,300 (83.9%); 14. Hernia of abdominal cavity 30,100 (84.7%); 15. Repair of recurrent inguinal hernia 28,400 (85.5%); 16. Primary repair of inguinal hernia NOS 26,700 (86.3%); 17. Simple umbilical hernia 26,300 (87.0%); 18. H/O: abdominal hernia 25,000 (87.7%); 19. Inguinal herniotomy 24,100 (88.4%); 20. Primary repair of femoral hernia 23,600 (89.0%); 21. Uncomplicated inguinal hernia 21,900 (89.7%)
Alcohol abuse	1. Alcohol dependence syndrome 1,142,800 (34.5%); 2. Alcohol problem drinking 847,100 (60.1%); 3. Alcoholic cirrhosis of liver 329,200 (70.0%); 4. Alcohol detoxification 167,200 (75.1%); 5. Alcoholism 155,400 (79.8%); 6. Alcohol dependence syndrome NOS 109,200 (83.1%); 7. Alcohol withdrawal syndrome 88,300 (85.7%); 8. Alcohol dependence 70,000 (87.8%); 9. Chronic alcoholism 62,800 (89.7%)
Allergic Conjunctivitis	1. Atopic conjunctivitis 586,100 (82.3%); 2. Chronic allergic conjunctivitis 76,200 (93.1%); 3. Acute allergic conjunctivitis 30,100 (97.3%); 4. Acute atopic conjunctivitis 12,800 (99.1%); 5. Seasonal allergic conjunctivitis 4,000 (99.6%)
Allergic Rhinitis	1. Hay fever - pollens 2,515,300 (30.7%); 2. Allergic rhinitis 1,339,900 (47.0%); 3. Hay fever 1,126,700 (60.8%); 4. Allergic rhinitis due to allergen 676,900 (69.0%); 5. Allergic rhinitis due to pollens 588,800 (76.2%); 6. H/O: hay fever 583,200 (83.3%); 7. Allergic rhinitis due to pollen 253,100 (86.4%); 8. Perennial allergic rhinitis 247,600 (89.5%)
Alopecia Areata	1. Alopecia areata 206,300 (95.9%); 2. [X]Other alopecia areata 8,700 (100.0%); 3. Ophiasis 0 (100.0%); 4. Ophiasis 0 (100.0%)
Alzheimer's dementia	1. Alzheimer's disease 803,600 (54.6%); 2. [X]Dementia in Alzheimer's disease 324,400 (76.6%); 3. [X]Dementia in Alzheimer's dis, atypical or mixed type 118,600 (84.6%); 4. Dementia in Alzheimer's disease with late onset 56,300 (88.5%); 5. [X]Alzheimer's dementia unspec 54,500 (92.2%)
Anxiety	1. Mixed anxiety and depressive disorder 18,609,100 (55.2%); 2. Anxiety disorder 6,194,400 (73.6%); 3. Anxiety state 1,161,500 (77.0%); 4. Panic attack 922,700 (79.7%); 5. [X]Mixed anxiety and depressive disorder 706,800 (81.8%); 6. Anxiousness 684,900 (83.9%); 7. Generalised anxiety disorder 7 item score 631,700 (85.7%); 8. Anxiousness - symptom 603,400 (87.5%); 9. [X]Anxiety NOS 550,600 (89.2%)
Appendicitis	1. Appendicectomy 415,900 (43.3%); 2. Emergency appendicectomy 164,800 (60.4%); 3. Acute appendicitis 105,200 (71.4%); 4. Emergency appendicectomy NEC 38,000 (75.3%); 5. Appendicitis 30,700 (78.5%); 6. Excision of appendix 29,500 (81.6%); 7. Emergency excision of appendix 24,200 (84.1%); 8. Laparoscopic appendicectomy 22,500 (86.4%); 9. Other excision of appendix NOS 20,700 (88.6%)
Asthma	1. Asthma 20,390,400 (15.8%); 2. Asthma annual review 12,636,600 (25.6%); 3. Asthma not disturbing sleep 8,515,200 (32.2%); 4. Asthma not limiting activities 8,182,100 (38.6%); 5. Asthma management 5,969,400 (43.2%); 6. Asthma medication review 5,688,400 (47.6%); 7. Asthma never causes daytime symptoms 4,757,100 (51.3%); 8. Asthma monitoring check done 4,690,400 (55.0%); 9. Asthma monitoring call first letter 4,570,900 (58.5%); 10. Asthma monitoring by nurse 2,183,300 (60.2%); 11. Asthma causes daytime symptoms 1 to 2 times per month 2,129,400 (61.8%); 12. Asthma causes daytime symptoms most days 2,058,600 (63.4%); 13. Asthma causes daytime symptoms 1 to 2 times per week 1,984,300 (65.0%); 14. Asthma self-management plan agreed 1,984,000 (66.5%); 15. Asthma control test score 1,942,500 (68.0%); 16. Asthma monitoring call second letter 1,910,900 (69.5%); 17. Number of asthma exacerbations in past year 1,792,700 (70.9%); 18. Acute exacerbation of asthma 1,725,400 (72.2%); 19. Asthma never disturbs sleep 1,719,300 (73.6%); 20. Asthma monitoring 1,534,400 (74.8%); 21. Asthma limiting activities 1,496,200 (75.9%); 22. Asthma daytime symptoms 1,465,700 (77.1%); 23. Asthma never restricts exercise 1,365,300 (78.1%); 24. Asthma follow-up 1,362,900 (79.2%); 25. Asthma sometimes restricts exercise 1,332,800 (80.2%); 26. Asthma disturbing sleep 1,295,800 (81.2%); 27. Emergency asthma patient visit since last encounter 1,247,600 (82.2%); 28. Asthma monitoring call third letter 1,052,600 (83.0%); 29. Asthma control step 2 1,039,200 (83.8%); 30. H/O: asthma 1,009,900 (84.6%); 31. Asthma monitoring invitation SMS (short message service) text message 973,200 (85.3%); 32. Asthma monitoring by doctor 893,600 (86.0%); 33. Excepted from asthma quality indicators - informed dissent 892,100 (86.7%); 34. Asthma treatment compliance satisfactory 824,700 (87.4%); 35. Health education - asthma 728,000 (87.9%); 36. Asthma control step 3 688,000 (88.5%); 37. Asthma self-management plan review 567,000 (88.9%); 38. Asthma NOS 521,100 (89.3%); 39. Patient has a written asthma personal action plan 490,700 (89.7%)
Autism	1. Autistic spectrum disorder 178,300 (24.9%); 2. Asperger's syndrome 163,300 (47.7%); 3. Autism 107,500 (62.6%); 4. Autism spectrum disorder 93,600 (75.7%); 5. Autistic disorder 78,300 (86.6%)
Autoimmune liver disease	1. Primary biliary cirrhosis 167,000 (53.3%); 2. Autoimmune hepatitis 98,700 (84.7%); 3. Primary sclerosing cholangitis 32,600 (95.1%); 4. Autoimmune chronic active hepatitis 15,000 (99.9%); 5. Primary biliary cholangitis 300 (100.0%)
Barrett’s oesophagus	1. Barrett's oesophagus 691,000 (71.3%); 2. Barrett's oesophagus 255,400 (97.6%); 3. Barrett's ulcer of oesophagus 22,000 (99.9%); 4. Barrett esophagus 400 (99.9%); 5. Barretts esophagus 300 (100.0%)

Outcome	Numbered list of the most commonly occurring codes with rounded number of occurrences and cumulative percentage of all occurrences
Breast cancer	1. Malignant neoplasm of female breast 3,077,800 (70.7%); 2. Ca female breast 542,100 (83.2%); 3. Malignant neoplasm of female breast NOS 204,100 (87.9%); 4. [RFC] Breast cancer 76,600 (89.6%); 5. Malignant tumour of breast 69,400 (91.2%)
CNS cancers	1. Malignant neoplasm of brain 115,600 (16.8%); 2. Cerebral meningioma 62,900 (26.0%); 3. Glioblastoma 61,300 (34.9%); 4. Glioblastoma multiforme 54,700 (42.8%); 5. Schwannoma 49,200 (50.0%); 6. [M]Astrocytoma NOS 34,400 (55.0%); 7. Secondary malignant neoplasm of brain 32,200 (59.7%); 8. Meningioma 30,200 (64.1%); 9. [M]Glioma NOS 20,200 (67.0%); 10. Malignant glioma 19,800 (69.9%); 11. Oligodendroglioma - category 15,400 (72.1%); 12. Malignant neoplasm of brain NOS 13,700 (74.1%); 13. Anaplastic astrocytoma 12,700 (76.0%); 14. [M]Gliomas 12,600 (77.8%); 15. [M]Glioma NOS 12,200 (79.6%); 16. Ependymoma - category 11,400 (81.2%); 17. Pilocytic astrocytoma 10,100 (82.7%); 18. Medulloblastoma 8,000 (83.9%); 19. Malignant neoplasm of frontal lobe 6,700 (84.9%); 20. [M]Meningioma NOS 6,600 (85.8%); 21. Cerebral tumour - malignant 5,700 (86.6%); 22. Astrocytoma 5,400 (87.4%); 23. Anaplastic oligodendroglioma 5,100 (88.2%); 24. Secondary malignant neoplasm of brain and spinal cord 4,700 (88.8%); 25. Paraganglioma 4,400 (89.5%)
COPD	1. Chronic obstructive pulmonary disease 5,454,200 (23.1%); 2. Chronic obstructive pulmonary disease annual review 4,009,500 (40.1%); 3. Number of chronic obstructive pulmonary disease exacerbations in past year 1,809,600 (47.8%); 4. Chronic obstructive pulmonary disease monitoring 1,290,000 (53.3%); 5. Chronic obstructive pulmonary disease monitoring first letter 1,167,000 (58.2%); 6. Acute exacerbation of chronic obstructive airways disease 1,082,100 (62.8%); 7. Chronic obstructive pulmonary disease assessment test score 959,000 (66.9%); 8. Moderate chronic obstructive pulmonary disease 954,600 (70.9%); 9. COPD self-management plan given 850,700 (74.5%); 10. Mild chronic obstructive pulmonary disease 756,300 (77.7%); 11. Severe chronic obstructive pulmonary disease 536,100 (80.0%); 12. Chronic obstructive airway disease 416,100 (81.8%); 13. Chronic obstructive pulmonary disease monitoring second letter 367,900 (83.3%); 14. Chronic obstructive lung disease 294,600 (84.6%); 15. Issue of chronic obstructive pulmonary disease rescue pack 257,200 (85.7%); 16. COPD medication review 228,300 (86.6%); 17. Chronic obstructive pulmonary disease self-management plan agreed 183,400 (87.4%); 18. Chronic obstructive pulmonary disease follow-up 180,800 (88.2%); 19. COAD - Chronic obstructive airways disease 171,100 (88.9%); 20. Chronic obstructive pulmonary disease clinical management plan 165,700 (89.6%)
Cholecystitis	1. Acute cholecystitis 202,600 (58.8%); 2. Cholecystitis 82,600 (82.8%); 3. Chronic cholecystitis 30,700 (91.7%); 4. Gallbladder calculus with acute cholecystitis 7,900 (94.0%); 5. Empyema of gallbladder 4,100 (95.1%)
Cigarette smoking	1. Smoking cessation advice 43,453,800 (35.8%); 2. Cigarette smoker 21,309,600 (53.4%); 3. Smoking cessation education 14,267,400 (65.2%); 4. Current smoker 13,615,700 (76.4%); 5. Moderate cigarette smoker (10-19 cigs/day) 2,686,300 (78.6%); 6. Trying to give up smoking 2,300,000 (80.5%); 7. Light cigarette smoker (1-9 cigs/day) 2,235,200 (82.3%); 8. Tobacco smoking consumption 1,505,600 (83.6%); 9. Rolls own cigarettes 1,283,200 (84.6%); 10. Seen by smoking cessation advisor 1,214,600 (85.6%); 11. Heavy cigarette smoker (20-39 cigs/day) 1,136,600 (86.6%); 12. Referral to smoking cessation advisor 1,086,400 (87.5%); 13. Not interested in stopping smoking 1,034,300 (88.3%); 14. Smoking cessation advice declined 999,600 (89.2%); 15. Nicotine replacement therapy 909,800 (89.9%)
Coeliac disease	1. Coeliac disease 544,700 (91.2%); 2. Gluten intolerance 21,800 (94.8%); 3. Coeliac disease NOS 9,400 (96.4%); 4. Coeliac disease annual review 6,500 (97.5%); 5. Coeliac disease monitoring 3,900 (98.1%)
Coronary artery disease	1. Ischaemic heart disease 4,902,100 (32.2%); 2. Framingham coronary heart disease 10 year risk score 2,542,400 (49.0%); 3. Coronary heart disease annual review 2,185,000 (63.3%); 4. Coronary heart disease monitoring 1st letter 1,645,700 (74.1%); 5. IHD - Ischaemic heart disease 1,238,400 (82.3%); 6. Primary prevention of ischaemic heart disease 395,100 (84.9%); 7. Coronary heart disease monitoring 2nd letter 381,200 (87.4%); 8. Coronary heart disease risk 236,300 (88.9%)
Crohn's disease	1. Crohn's disease 739,600 (70.6%); 2. Crohn's regional enteritis 207,900 (90.5%); 3. CC - Crohn's colitis 40,900 (94.4%); 4. Crohn's disease of terminal ileum 16,200 (95.9%); 5. Regional enteritis - Crohn 11,700 (97.0%)
Cutaneous warts	1. Verruca plantaris 1,659,100 (24.6%); 2. Viral wart 1,216,000 (42.6%); 3. Plantar wart 767,500 (54.0%); 4. Hand wart 577,800 (62.5%); 5. Seborrhoeic wart 370,300 (68.0%); 6. Plain wart 346,900 (73.1%); 7. Seborrhoeic wart 295,800 (77.5%); 8. Seborrhoeic wart 274,400 (81.6%); 9. Genital warts 260,700 (85.4%); 10. Verruca vulgaris 206,000 (88.5%)
Depression	1. Mixed anxiety and depressive disorder 18,609,100 (33.1%); 2. Depression 5,639,500 (43.1%); 3. Low mood 5,469,100 (52.9%); 4. Depressive disorder 4,668,000 (61.2%); 5. Depression interim review 3,236,600 (66.9%); 6. Depressed 2,810,500 (71.9%); 7. Depressed mood 1,558,600 (74.7%); 8. Depression medication review 1,268,400 (77.0%); 9. H/O: depression 1,255,900 (79.2%); 10. Depressive episode 966,600 (80.9%); 11. [X]Moderate depressive episode 781,100 (82.3%); 12. [X]Mixed anxiety and depressive disorder 706,800 (83.6%); 13. Reactive depression (situational) 678,400 (84.8%); 14. [X]Depressive episode, unspecified 642,200 (85.9%); 15. Postpartum depression 573,500 (86.9%); 16. Symptoms of depression 502,700 (87.8%); 17. C/O - feeling depressed 485,900 (88.7%); 18. Depressive episode 425,700 (89.5%)
Dermatophyte infection	1. Fungal nail infection 1,473,100 (27.0%); 2. Athlete's foot 608,000 (38.1%); 3. Dermatophytosis 458,300 (46.5%); 4. Ringworm 448,000 (54.7%); 5. Dermatophytosis of nail 338,800 (60.9%); 6. Tinea pedis 337,000 (67.0%); 7. Tinea cruris 326,500 (73.0%); 8. Onychomycosis 289,600 (78.3%); 9. Tinea corporis 248,000 (82.9%); 10. Dermatophytosis of foot 230,100 (87.1%); 11. Dermatophytosis of the body 113,700 (89.1%)
Diabetes mellitus	1. Type 2 diabetes mellitus 22,645,700 (27.4%); 2. Diabetic annual review 8,575,500 (37.8%); 3. O/E - Right diabetic foot at low risk 7,472,900 (46.9%); 4. O/E - Left diabetic foot at low risk 7,433,600 (55.9%); 5. Diabetes mellitus 4,912,200 (61.9%); 6. Diabetic on oral treatment 4,810,900 (67.7%); 7. Type 1 diabetes mellitus 2,238,400 (70.4%); 8. Diabetic on diet only 1,698,500 (72.5%); 9. Type II diabetic dietary review 1,523,000 (74.3%); 10. Diabetic on insulin 1,379,800 (76.0%); 11. O/E - Right diabetic foot at moderate risk 1,323,500 (77.6%); 12. O/E - Left diabetic foot at

Outcome	Numbered list of the most commonly occurring codes with rounded number of occurrences and cumulative percentage of all occurrences
	moderate risk 1,316,700 (79.2%); 13. Agreeing on diabetes care plan 1,265,300 (80.7%); 14. Diabetes self-management plan agreed 1,142,400 (82.1%); 15. Background diabetic retinopathy 820,000 (83.1%); 16. Diabetes management plan given 763,600 (84.0%); 17. Diabetic foot examination 703,100 (84.9%); 18. Diabetes medication review 703,000 (85.7%); 19. Patient on maximal tolerated therapy for diabetes 655,900 (86.5%); 20. Non-insulin dependent diabetes mellitus 650,000 (87.3%); 21. Type II diabetes mellitus 620,400 (88.0%); 22. Insulin treated Type 2 diabetes mellitus 556,900 (88.7%); 23. O/E - right eye background diabetic retinopathy 537,600 (89.4%)
Diverticular disease	1. Diverticular disease 809,600 (31.4%); 2. Diverticulosis 745,800 (60.3%); 3. Diverticulitis 573,900 (82.5%); 4. Diverticula of intestine 152,100 (88.4%); 5. Diverticular disease of colon 132,500 (93.5%)
Dyslipidaemia	1. Pure hypercholesterolaemia 2,694,100 (33.9%); 2. Hyperlipidaemia 2,020,100 (59.4%); 3. Mixed hyperlipidaemia 560,800 (66.5%); 4. Lipid-lowering therapy 448,300 (72.1%); 5. Patient on maximal tolerated lipid lowering therapy 390,000 (77.0%); 6. Hyperlipidaemia screen 269,300 (80.4%); 7. Disorder of lipid metabolism 257,000 (83.6%); 8. Hypercholesterolaemia 244,400 (86.7%); 9. Serum lipids high 142,100 (88.5%)
Eosinophilic Oesophagitis	1. Eosinophilic oesophagitis 22,900 (99.9%); 2. Eosinophilic esophagitis 0 (100.0%)
Epilepsy	1. Epilepsy 2,319,900 (38.1%); 2. Epilepsy medication review 1,277,000 (59.1%); 3. Epilepsy monitoring 596,400 (68.9%); 4. Generalised epilepsy 201,800 (72.2%); 5. No epilepsy drug side effects 134,400 (74.4%); 6. Epilepsy NOS 118,100 (76.4%); 7. Temporal lobe epilepsy 110,400 (78.2%); 8. Contraceptive advice for patients with epilepsy 97,500 (79.8%); 9. Follow-up epilepsy assessment 89,000 (81.2%); 10. H/O: epilepsy 78,700 (82.5%); 11. Pre-conception advice for patients with epilepsy 67,700 (83.6%); 12. Pregnancy advice for patients with epilepsy 67,600 (84.8%); 13. Seen in epilepsy clinic 64,600 (85.8%); 14. Epilepsy monitoring call first letter 45,500 (86.6%); 15. Petit mal (minor) epilepsy 41,400 (87.3%); 16. Complex partial epileptic seizure 39,900 (87.9%); 17. Pregnancy advice for patients with epilepsy not indicated 35,600 (88.5%); 18. Epilepsy management plan given 34,700 (89.1%); 19. Contraceptive advice for patients with epilepsy not indicated 34,500 (89.6%)
Fatty liver	1. Non-alcoholic fatty liver 323,400 (47.0%); 2. Fatty liver 168,200 (71.5%); 3. Steatosis of liver 75,700 (82.5%); 4. Fatty change of liver 49,100 (89.6%); 5. Alcoholic fatty liver 44,600 (96.1%)
Fibrosis/sclerosis/cirrhosis	1. Alcoholic cirrhosis of liver 329,200 (29.9%); 2. Alcoholic liver damage 181,000 (46.4%); 3. Cirrhosis of liver 174,600 (62.2%); 4. Primary biliary cirrhosis 167,000 (77.4%); 5. Cirrhosis and chronic liver disease 105,300 (87.0%)
Food allergy	1. Nut allergy 224,800 (25.9%); 2. Food allergy 199,200 (48.9%); 3. Allergy to peanuts 140,000 (65.1%); 4. Allergy to eggs 122,100 (79.1%); 5. H/O: food allergy 71,600 (87.4%)
Gastritis and duodenitis	1. Gastritis 961,500 (39.5%); 2. Gastritis and duodenitis 387,800 (55.4%); 3. Acute gastritis 320,700 (68.5%); 4. Duodenitis 241,800 (78.5%); 5. [X]Other gastritis 117,900 (83.3%); 6. Helicobacter-associated gastritis 78,500 (86.5%); 7. Chronic gastritis 66,700 (89.3%)
Gastro oesophageal reflux	1. Reflux oesophagitis 2,436,800 (35.5%); 2. Gastro-oesophageal reflux 1,984,900 (64.4%); 3. Acid reflux 768,000 (75.6%); 4. Gastric reflux 349,200 (80.7%); 5. Oesophageal reflux 268,800 (84.6%); 6. Gastrooesophageal reflux disease 242,700 (88.2%)
Heart failure	1. Heart failure 920,900 (19.2%); 2. Congestive heart failure 679,200 (33.3%); 3. Left ventricular failure 668,000 (47.2%); 4. Congestive cardiac failure 488,500 (57.4%); 5. Left ventricular systolic dysfunction 279,100 (63.2%); 6. Echocardiogram shows left ventricular systolic dysfunction 184,100 (67.1%); 7. Heart failure annual review 181,200 (70.8%); 8. Seen in heart failure clinic 156,700 (74.1%); 9. Impaired left ventricular function 115,400 (76.5%); 10. Seen by community heart failure nurse 94,000 (78.5%); 11. Heart failure monitoring first letter 81,800 (80.2%); 12. Referral to heart failure clinic 62,700 (81.5%); 13. Heart failure 6 month review 60,400 (82.7%); 14. Pulmonary oedema 55,700 (83.9%); 15. Impaired left ventricular function 54,700 (85.0%); 16. Heart failure review completed 50,200 (86.1%); 17. Cardiac failure 37,500 (86.9%); 18. Left ventricular diastolic dysfunction 35,900 (87.6%); 19. Heart failure follow-up 35,100 (88.3%); 20. Echocardiogram shows left ventricular diastolic dysfunction 34,200 (89.0%); 21. Heart failure NOS 34,200 (89.8%)
Herpes simplex	1. Herpes simplex 418,000 (26.1%); 2. Cold sore (herpetic) 406,000 (51.4%); 3. Genital herpes simplex 277,200 (68.7%); 4. Herpes labialis 64,300 (72.8%); 5. O/E-herpes labialis-cold sore 61,800 (76.6%); 6. O/E - cold sore 53,900 (80.0%); 7. Herpetic gingivostomatitis 50,600 (83.1%); 8. Genital herpes unspecified 48,800 (86.2%); 9. Eczema herpeticum - Kaposi's varicelliform eruption 30,100 (88.1%); 10. Herpes simplex viral infection 28,200 (89.8%)
Hip fracture	1. Fracture of neck of femur 1,040,000 (66.9%); 2. Hip fracture 207,800 (80.3%); 3. Closed fracture of neck of femur 76,400 (85.2%); 4. Primary open reduction and internal fixation of proximal femoral fracture with screw/nail and plate device 43,400 (88.0%); 5. Primary open reduction of fracture of neck of femur and open fixation using dynamic hip screw 33,200 (90.1%)
Hodgkin lymphoma	1. Hodgkin's disease 110,900 (63.7%); 2. Hodgkin lymphoma 13,100 (71.2%); 3. Hodgkin's disease, nodular sclerosis 9,500 (76.7%); 4. Hodgkin's disease (clinical) 9,100 (81.9%); 5. Hodgkin lymphoma 5,000 (84.8%); 6. [M]Hodgkin's disease 4,200 (87.2%); 7. Hodgkin's disease NOS 2,300 (88.6%); 8. Hodgkin lymphoma, nodular sclerosis 2,300 (89.8%)
Hypertension	1. Essential hypertension 35,271,200 (61.1%); 2. Hypertensive disease 10,600,000 (79.4%); 3. Hypertension annual review 4,693,700 (87.5%); 4. Hypertension six month review 1,240,200 (89.7%); 5. Hypertension 969,300 (91.4%)
Impetigo	1. Impetigo 2,513,600 (93.8%); 2. Impetigo NOS 129,700 (98.6%); 3. Impetigo contagiosa unspecified 10,400 (99.0%); 4. Bullous impetigo 8,400 (99.3%); 5. Impetigo follicularis 5,500 (99.5%)

Outcome	Numbered list of the most commonly occurring codes with rounded number of occurrences and cumulative percentage of all occurrences
Irritable bowel syndrome	1. Irritable bowel syndrome 2,523,300 (72.6%); 2. Irritable colon - Irritable bowel syndrome 709,000 (93.0%); 3. Irritable colon 97,200 (95.8%); 4. Irritable bowel syndrome with diarrhoea 64,400 (97.7%); 5. Irritable bowel - IBS 57,500 (99.3%)
Lung cancer	1. Lung cancer 612,400 (33.4%); 2. Malignant tumour of lung 505,400 (61.0%); 3. Malignant neoplasm of lower respiratory tract 221,200 (73.1%); 4. Secondary malignant neoplasm of lung 67,400 (76.8%); 5. Primary malignant neoplasm of lung 65,600 (80.4%); 6. Malignant neoplasm of upper lobe of lung 53,700 (83.3%); 7. Malignant neoplasm of upper lobe, bronchus or lung 47,900 (85.9%); 8. Malignant neoplasm of main bronchus 40,700 (88.2%)
Melanoma	1. Malignant melanoma of skin 471,100 (36.5%); 2. Malignant neoplasm of skin 201,700 (52.1%); 3. Malignant melanoma 69,500 (57.5%); 4. Excision of melanoma 59,300 (62.0%); 5. Lentigo maligna 52,200 (66.1%); 6. Malignant melanoma of lower leg 26,000 (68.1%); 7. Malignant neoplasm of skin NOS 24,300 (70.0%); 8. Malignant melanoma of back 22,100 (71.7%); 9. History of primary malignant neoplasm of skin 18,700 (73.1%); 10. Superficial spreading melanoma 16,400 (74.4%); 11. Malignant neoplasm of skin of lower limb and hip 14,500 (75.5%); 12. H/O Malignant melanoma 14,100 (76.6%); 13. Melanoma in situ 12,500 (77.6%); 14. Melanoma in situ of skin 11,300 (78.5%); 15. Malignant melanoma of thigh 10,200 (79.2%); 16. Malignant melanoma of lower limb and hip 9,900 (80.0%); 17. Malignant melanoma of skin NOS 9,800 (80.8%); 18. Malignant neoplasm of skin of trunk 9,500 (81.5%); 19. [M]Lentigo maligna melanoma 9,500 (82.2%); 20. Malignant neoplasm of skin of nose (external) 8,200 (82.9%); 21. Malignant melanoma of skin of trunk 8,100 (83.5%); 22. Malignant melanoma of upper arm 7,700 (84.1%); 23. Malignant melanoma of eye 7,400 (84.7%); 24. Malignant neoplasm of skin of upper limb and shoulder 6,700 (85.2%); 25. Malignant neoplasm of skin of cheek, external 6,000 (85.7%); 26. Nodular melanoma 5,900 (86.1%); 27. Malignant neoplasm of scalp 5,800 (86.6%); 28. Malignant melanoma of upper limb 5,700 (87.0%); 29. Malignant neoplasm of skin of forehead 5,700 (87.4%); 30. Malignant melanoma of fore-arm 5,000 (87.8%); 31. Malignant neoplasm of skin of lower leg 4,800 (88.2%); 32. Malignant melanoma of choroid 4,700 (88.6%); 33. Malignant melanoma of breast 4,700 (88.9%); 34. Malignant melanoma of scalp and neck 4,000 (89.2%); 35. Malignant melanoma of neck 3,800 (89.5%); 36. Malignant neoplasm of skin of temple 3,800 (89.8%)
Metabolic syndrome	1. Cardiac syndrome X 18,800 (56.5%); 2. Metabolic syndrome 10,900 (89.2%); 3. Metabolic syndrome X 3,300 (98.9%); 4. Reaven's syndrome 300 (99.9%); 5. Reaven's syndrome 100 (100.0%)
Migraine	1. Migraine 4,230,600 (76.7%); 2. H/O: migraine 352,900 (83.1%); 3. Migraine with typical aura 299,900 (88.6%); 4. Migraine NOS 190,400 (92.0%); 5. Migraine with aura 67,900 (93.3%)
Molluscum contagiosum	1. Molluscum contagiosum infection 1,070,300 (99.0%); 2. Molluscum contagiosum infection of eyelid 5,800 (99.5%); 3. [SHHAPT] Molluscum contagiosum 4,900 (100.0%); 4. Genital Molluscum contagiosum 0 (100.0%); 5. Molluscum Contagiosum 0 (100.0%)
Multiple sclerosis	1. Multiple sclerosis 768,900 (87.6%); 2. [RFC] Multiple sclerosis 24,400 (90.4%); 3. Relapsing remitting multiple sclerosis 15,800 (92.2%); 4. Multiple sclerosis NOS 15,600 (93.9%); 5. Multiple sclerosis - relapsing remitting 12,000 (95.3%)
Myeloma	1. Multiple myeloma 367,400 (70.1%); 2. [M]Myeloma NOS 89,100 (87.1%); 3. Plasmacytoma 15,100 (90.0%); 4. [M]Plasma cell myeloma 10,900 (92.0%); 5. Lambda light chain myeloma 10,400 (94.0%)
Myocardial infarction	1. Acute myocardial infarction 1,389,800 (49.9%); 2. Acute non-ST segment elevation myocardial infarction 487,700 (67.5%); 3. MI - acute myocardial infarction 281,900 (77.6%); 4. Acute ST segment elevation myocardial infarction 203,700 (84.9%); 5. Acute myocardial infarction of inferior wall 86,900 (88.0%); 6. Old myocardial infarction 50,800 (89.8%)
Non-Hodgkin lymphoma	1. Non-Hodgkin's lymphoma 208,300 (23.9%); 2. [M]Lymphoma NOS 104,500 (35.9%); 3. Diffuse large B-cell lymphoma 50,800 (41.7%); 4. [M]Non-Hodgkin's lymphoma 46,300 (47.0%); 5. Follicular lymphoma NOS 45,200 (52.2%); 6. B-cell non-Hodgkin's lymphoma 39,900 (56.8%); 7. Follicular non-Hodgkin's lymphoma 33,500 (60.6%); 8. Malignant lymphoma 32,700 (64.3%); 9. Malignant neoplasm lymphatic or haematopoietic tissue NOS 28,300 (67.6%); 10. Non hodgkin lymphoma 25,800 (70.5%); 11. Non-Hodgkin's lymphoma (clinical) 21,300 (73.0%); 12. Malignant lymphoma (clinical) 17,200 (74.9%); 13. Mantle cell lymphoma 17,000 (76.9%); 14. Malignant lymphoma 16,300 (78.8%); 15. Non-Hodgkin lymphoma (category) 13,200 (80.3%); 16. [M]Malignant lymphoma, non-Hodgkin's type 12,500 (81.7%); 17. [M] Cutaneous lymphoma 7,700 (82.6%); 18. Follicular lymphoma 7,700 (83.5%); 19. Suspected lymphoma 7,200 (84.3%); 20. Burkitt's lymphoma 7,100 (85.1%); 21. [X]Non-Hodgkin's lymphoma NOS 5,800 (85.8%); 22. Low grade B-cell lymphoma 5,300 (86.4%); 23. [M]Lymphocytic lymphoma NOS 5,200 (87.0%); 24. Malignant lymphoma NOS 5,200 (87.6%); 25. Monocytoid B-cell lymphoma 4,800 (88.1%); 26. [M]Malignant lymphoma, lymphoplasmacytoid type 4,400 (88.6%); 27. [M]Lymphomas, NOS or diffuse 4,200 (89.1%); 28. Peripheral T-cell lymphoma 4,000 (89.6%); 29. Cutaneous T-cell lymphoma 3,500 (90.0%)
Nonmelanoma skin cancer	1. Basal cell carcinoma of skin 2,654,800 (56.1%); 2. Squamous cell carcinoma - category 585,100 (68.5%); 3. Bowen's disease of skin 404,700 (77.0%); 4. Squamous cell carcinoma of skin 314,100 (83.7%); 5. Basal cell carcinoma 235,000 (88.7%)
Obesity	1. Obesity 1,901,800 (35.1%); 2. Body mass index 30+ - obesity 1,802,300 (68.5%); 3. Obesity monitoring 502,000 (77.7%); 4. Intervention for risk to health associated with overweight and obesity, general advice on healthy weight and lifestyle 260,400 (82.5%); 5. Morbid obesity 153,100 (85.4%); 6. Follow-up obesity assessment 149,200 (88.1%)
Oesophagitis	1. Reflux oesophagitis 2,436,800 (45.0%); 2. Oesophagitis 1,320,100 (69.4%); 3. Barrett's oesophagus 691,000 (82.1%); 4. Barrett's oesophagus 255,400 (86.9%); 5. Gastro-oesophageal reflux disease without oesophagitis 229,400 (91.1%)

Outcome	Numbered list of the most commonly occurring codes with rounded number of occurrences and cumulative percentage of all occurrences
Oesophageal varices	1. Oesophageal varices 88,400 (54.2%); 2. Oesophageal varices with bleeding 26,400 (70.4%); 3. Oesophageal varices NOS 15,300 (79.7%); 4. Fibreoptic endoscopic banding of oesophageal varices 11,700 (86.9%); 5. Oesophageal varices in alcoholic cirrhosis of the liver 7,400 (91.5%)
Osteoporosis	1. Osteoporosis 2,565,700 (77.7%); 2. Pathological fracture due to osteoporosis 99,400 (80.7%); 3. Health education - osteoporosis 88,900 (83.4%); 4. Osteoporotic vertebral collapse 58,500 (85.1%); 5. Lumbar DXA scan result osteoporotic 46,300 (86.5%); 6. Seen in osteoporosis clinic 43,900 (87.9%); 7. Referral to osteoporosis clinic 30,100 (88.8%); 8. Osteoporosis NOS 28,300 (89.6%)
Pancreatic cancer	1. Malignant tumour of pancreas 289,700 (67.5%); 2. Malignant tumour of head of pancreas 51,700 (79.5%); 3. [M]Pancreatic adenomas and carcinomas 25,000 (85.4%); 4. Malignant neoplasm of pancreas NOS 23,200 (90.8%); 5. [M]Pancreatic adenoma or carcinoma NOS 12,300 (93.6%)
Pancreatitis	1. Acute pancreatitis 187,400 (41.0%); 2. Chronic pancreatitis 123,800 (68.1%); 3. Pancreatitis 71,700 (83.8%); 4. Gallstone acute pancreatitis 25,300 (89.3%); 5. Alcohol-induced chronic pancreatitis 24,100 (94.6%)
Parkinson's disease	1. Parkinson's disease 1,141,900 (82.7%); 2. Dementia in Parkinsons disease 80,300 (88.5%); 3. Parkinson's disease NOS 69,900 (93.6%); 4. Seen by Parkinson's disease service 23,200 (95.3%); 5. Secondary parkinsonism 11,900 (96.1%)
Pelvis fracture	1. Closed fracture pelvis, single pubic ramus 119,700 (25.4%); 2. Fracture or disruption of pelvis 48,400 (35.7%); 3. Closed fracture of pelvis 48,100 (45.9%); 4. Fracture of acetabulum 33,700 (53.1%); 5. Closed fracture pubis 33,300 (60.2%); 6. Closed fracture pelvis, multiple pubic rami - stable 31,900 (66.9%); 7. Fracture of pubis 28,000 (72.9%); 8. Fracture of coccyx 22,000 (77.6%); 9. Fracture of pubic rami 20,300 (81.9%); 10. Fracture of sacrum 14,600 (85.0%); 11. Closed fracture acetabulum 10,200 (87.1%); 12. Closed fracture sacrum 9,400 (89.1%)
Peptic ulcer disease	1. Duodenal ulcer 308,100 (33.0%); 2. Gastric ulcer 202,000 (54.7%); 3. H/O: peptic ulcer 40,700 (59.0%); 4. Duodenal ulcer NOS 39,600 (63.3%); 5. Peptic ulcer 38,500 (67.4%); 6. Gastric erosions 37,200 (71.4%); 7. Gastric ulcer NOS 34,700 (75.1%); 8. Peptic ulcer NOS 29,400 (78.3%); 9. Peptic ulcer symptoms 16,200 (80.0%); 10. Chronic duodenal ulcer NOS 12,600 (81.3%); 11. Acute duodenal ulcer with haemorrhage 12,400 (82.7%); 12. Acute duodenal ulcer with perforation 11,100 (83.9%); 13. H/O: duodenal ulcer 10,900 (85.0%); 14. Duodenal erosion 9,000 (86.0%); 15. Closure of perforated duodenal ulcer 8,500 (86.9%); 16. Chronic duodenal ulcer 7,800 (87.7%); 17. Duodenal ulcer disease 7,600 (88.5%); 18. H/O: gastric ulcer 7,500 (89.3%)
Peripheral artery disease	1. Intermittent claudication 437,900 (38.0%); 2. Raynaud's phenomenon 166,200 (52.4%); 3. Peripheral vascular disease NOS 98,700 (60.9%); 4. Claudication 60,000 (66.1%); 5. Raynaud's disease 46,800 (70.2%); 6. Mixed diabetic ulcer - foot 45,000 (74.1%); 7. Percutaneous transluminal angioplasty of femoral artery 35,700 (77.2%); 8. Ischaemic ulcer diabetic foot 29,700 (79.8%); 9. Ischaemic leg 28,200 (82.2%); 10. Percutaneous transluminal angioplasty of iliac artery 18,400 (83.8%); 11. Gangrene of toe 17,000 (85.3%); 12. Femoral endarterectomy 13,800 (86.5%); 13. Peripheral ischaemic vascular disease 12,900 (87.6%); 14. Percutaneous angioplasty of popliteal artery 8,600 (88.3%); 15. Peripheral ischaemia 8,600 (89.1%); 16. Diabetic peripheral angiopathy 6,000 (89.6%)
Peripheral neuropathies	1. Sciatica 3,168,700 (39.2%); 2. Carpal tunnel syndrome 1,502,900 (57.8%); 3. Lumbago with sciatica 699,600 (66.5%); 4. Peripheral neuropathy 353,700 (70.8%); 5. Bell's palsy 309,500 (74.7%); 6. Trigeminal neuralgia NOS 277,500 (78.1%); 7. CTS - Carpal tunnel syndrome 179,200 (80.3%); 8. Meralgia paraesthetica 107,900 (81.6%); 9. Trigeminal neuralgia 99,500 (82.9%); 10. Morton's metatarsalgia 91,900 (84.0%); 11. Acute vestibular neuronitis 79,500 (85.0%); 12. Brachial (cervical) neuritis 70,700 (85.9%); 13. Lumbar disc prolapse with radiculopathy 68,500 (86.7%); 14. Peripheral nerve disease 54,500 (87.4%); 15. Ulnar nerve entrapment 53,000 (88.0%); 16. Radiculopathy 51,300 (88.7%); 17. Ulnar neuropathy 38,800 (89.2%); 18. Cauda equina syndrome 36,400 (89.6%)
Peritonitis	1. Peritonitis 18,900 (14.7%); 2. Perforated diverticulum 17,700 (28.4%); 3. Acute duodenal ulcer with perforation 11,100 (37.1%); 4. Acute appendicitis with peritonitis 9,000 (44.1%); 5. Closure of perforated duodenal ulcer 8,500 (50.7%); 6. Perforated diverticulum of colon 8,500 (57.3%); 7. Acute gangrenous appendicitis 7,500 (63.1%); 8. Perforated chronic duodenal ulcer 5,600 (67.5%); 9. Acute gastric ulcer with perforation 2,800 (69.6%); 10. Duodenal ulcer with perforation 2,500 (71.6%); 11. Chronic duodenal ulcer with perforation 2,200 (73.3%); 12. Closure of perforated gastric ulcer 2,000 (74.9%); 13. Perforated diverticulum of intestine 1,800 (76.2%); 14. Subphrenic abscess 1,700 (77.6%); 15. Acute appendicitis without peritonitis 1,700 (78.9%); 16. Faecal peritonitis 1,600 (80.1%); 17. Acute peptic ulcer with perforation 1,600 (81.4%); 18. Retroperitoneal abscess 1,500 (82.5%); 19. Drainage of intraperitoneal abscess 1,200 (83.5%); 20. Acute peritonitis 1,200 (84.4%); 21. Spontaneous bacterial peritonitis 1,200 (85.4%); 22. Perforated diverticulum of large intestine 1,200 (86.3%); 23. Perforated chronic gastric ulcer 1,100 (87.1%); 24. Peritonitis - bacterial 1,100 (88.0%); 25. Peritonitis NOS 700 (88.6%); 26. Diverticular disease of both small and large intestine with perforation and abscess 700 (89.1%); 27. Peritoneal dialysis-associated peritonitis 700 (89.6%)
Prostate cancer	1. Malignant tumour of prostate 4,324,100 (93.4%); 2. QCancer prostate cancer risk 80,200 (95.2%); 3. [RFC] Cancer of the prostate 51,200 (96.3%); 4. Gleason prostate grade 5-7 (medium) 36,800 (97.1%); 5. Gleason grade finding for prostatic cancer 29,000 (97.7%)
Spine fracture	1. Fracture of lumbar vertebra 114,800 (13.4%); 2. Fracture of thoracic vertebra 98,200 (24.8%); 3. Closed fracture thoracic vertebra, wedge 86,500 (34.9%); 4. Closed fracture lumbar vertebra, wedge 67,300 (42.7%); 5. Closed fracture lumbar vertebra 63,000 (50.1%); 6. H/O: vertebral fracture 58,800 (56.9%); 7. Closed fracture of cervical spine 41,400 (61.8%); 8. Closed fracture thoracic vertebra 38,000 (66.2%); 9. Fracture of spine without mention of spinal cord injury 24,800 (69.1%); 10. Osteoporosis with pathological fracture of thoracic vertebrae 20,400 (71.5%); 11. Osteoporosis with pathological fracture of lumbar vertebrae 18,800 (73.7%); 12. Closed fracture of vertebral column 18,200 (75.8%); 13. Fracture of vertebra without spinal cord lesion 15,200 (77.6%); 14. Fracture of lumbar spine 14,500 (79.3%); 15. Fracture of cervical spine 11,600 (80.6%); 16. Fracture of thoracic spine 11,500 (81.9%); 17. Multiple fractures of thoracic spine 10,800 (83.2%); 18. Closed fracture axis, odontoid process 8,800 (84.2%); 19. Closed fracture lumbar

Outcome	Numbered list of the most commonly occurring codes with rounded number of occurrences and cumulative percentage of all occurrences
	vertebra, transverse process 5,600 (84.9%); 20. Fracture of second cervical vertebra 4,600 (85.4%); 21. Balloon kyphoplasty of fracture of spine 4,600 (85.9%); 22. Multiple fractures of cervical spine 4,300 (86.4%); 23. Fracture of spine with spinal cord lesion 4,200 (86.9%); 24. Closed multiple fractures of thoracic spine 4,200 (87.4%); 25. Fatigue fracture of vertebra 4,100 (87.9%); 26. Fracture of vertebral column 4,000 (88.4%); 27. Vertebroplasty of fracture of spine 3,600 (88.8%); 28. Closed fracture lumbar vertebra, burst 3,600 (89.2%); 29. Fracture of spine without mention of spinal cord lesion NOS 3,500 (89.6%); 30. Fixation of spinal fracture 3,300 (90.0%)
Stroke	1. Stroke monitoring 506,000 (19.8%); 2. Stroke/transient ischaemic attack monitoring first letter 404,900 (35.6%); 3. Seen in stroke clinic 232,700 (44.7%); 4. Cerebral infarction 184,100 (51.9%); 5. Stroke / transient ischaemic attack referral 160,500 (58.2%); 6. CVA - cerebrovascular accident due to cerebral artery occlusion 137,100 (63.6%); 7. Stroke/transient ischaemic attack monitoring second letter 116,400 (68.1%); 8. Cerebral arterial occlusion 112,700 (72.5%); 9. Stroke unspecified 92,200 (76.1%); 10. Referral to stroke clinic 90,600 (79.7%); 11. Stroke/transient ischaemic attack monitoring third letter 43,600 (81.4%); 12. Stroke 36,100 (82.8%); 13. Cerebellar infarction 35,600 (84.2%); 14. H/O: stroke 32,600 (85.5%); 15. Left sided cerebral infarction 32,400 (86.7%); 16. Stroke due to cerebral arterial occlusion 29,300 (87.9%); 17. [RFC] Stroke 28,400 (89.0%)
Thromboembolic diseases	1. Deep venous thrombosis 738,400 (43.9%); 2. Deep vein thrombosis 187,000 (55.1%); 3. Phlebitis and thrombophlebitis 156,400 (64.4%); 4. Deep vein phlebitis and thrombophlebitis of the leg 149,200 (73.3%); 5. Thrombophlebitis 71,500 (77.5%); 6. H/O: Deep Vein Thrombosis 56,600 (80.9%); 7. Suspected deep vein thrombosis 48,600 (83.8%); 8. Deep vein thrombosis of lower limb 43,800 (86.4%); 9. Referral to deep vein thrombosis clinic 38,700 (88.7%); 10. Portal vein thrombosis 13,100 (89.5%)
Ulcerative colitis	1. Ulcerative colitis 920,000 (79.7%); 2. Ulcerative colitis and/or proctitis 116,200 (89.8%); 3. Ulcerative proctitis 35,200 (92.8%); 4. Ulcerative proctocolitis 34,000 (95.8%); 5. H/O: ulcerative colitis 17,500 (97.3%)
Urticaria	1. Urticaria 1,496,100 (63.5%); 2. Allergic urticaria 304,700 (76.4%); 3. Urticaria NOS 116,000 (81.3%); 4. Angioneurotic oedema 115,700 (86.2%); 5. Idiopathic urticaria 86,700 (89.9%)
Vascular dementia	1. Vascular dementia 600,600 (91.5%); 2. [X]Vascular dementia, unspecified 23,400 (95.0%); 3. Mixed cortical and subcortical vascular dementia 10,900 (96.7%); 4. VAD - Vascular dementia 7,000 (97.8%); 5. Subcortical vascular dementia 6,000 (98.7%)
Wrist fracture	1. Closed fracture of distal end of radius 811,500 (17.9%); 2. Fracture of metacarpal bone 806,600 (35.8%); 3. Closed fracture of wrist 608,200 (49.2%); 4. Fracture of scaphoid bone of wrist 392,000 (57.9%); 5. Closed Colles' fracture 344,800 (65.5%); 6. Closed fracture of the scaphoid 217,000 (70.3%); 7. Closed fracture of metacarpal bone(s) 115,700 (72.8%); 8. Hand fracture - metacarpal bone 115,200 (75.4%); 9. Closed fracture finger metacarpal 106,400 (77.7%); 10. Fracture at wrist and hand level 83,700 (79.6%); 11. Fracture of other metacarpal bone 76,600 (81.3%); 12. Greenstick fracture of distal radius 73,900 (82.9%); 13. Closed fracture radial styloid 70,900 (84.5%); 14. Closed fracture radius and ulna, distal 66,700 (85.9%); 15. Closed reduction of fracture of wrist 46,900 (87.0%); 16. Closed fracture finger metacarpal neck 42,600 (87.9%); 17. Fracture of forearm 41,900 (88.9%); 18. Closed fracture navicular 37,900 (89.7%)

Supplementary Table 5: Numbered lists of the most commonly occurring codes with rounded number of occurrences and cumulative percentage of all occurrences (e.g., the code "Attention deficit hyperactivity disorder" makes up 72.5% of all codes for the ADHD outcome; the codes "Attention deficit hyperactivity disorder" and "Attention deficit with hyperactivity" together make up 79.9% of all codes for the ADHD outcome, etc…)

Supplementary Figure 1: Comparison with previous studies

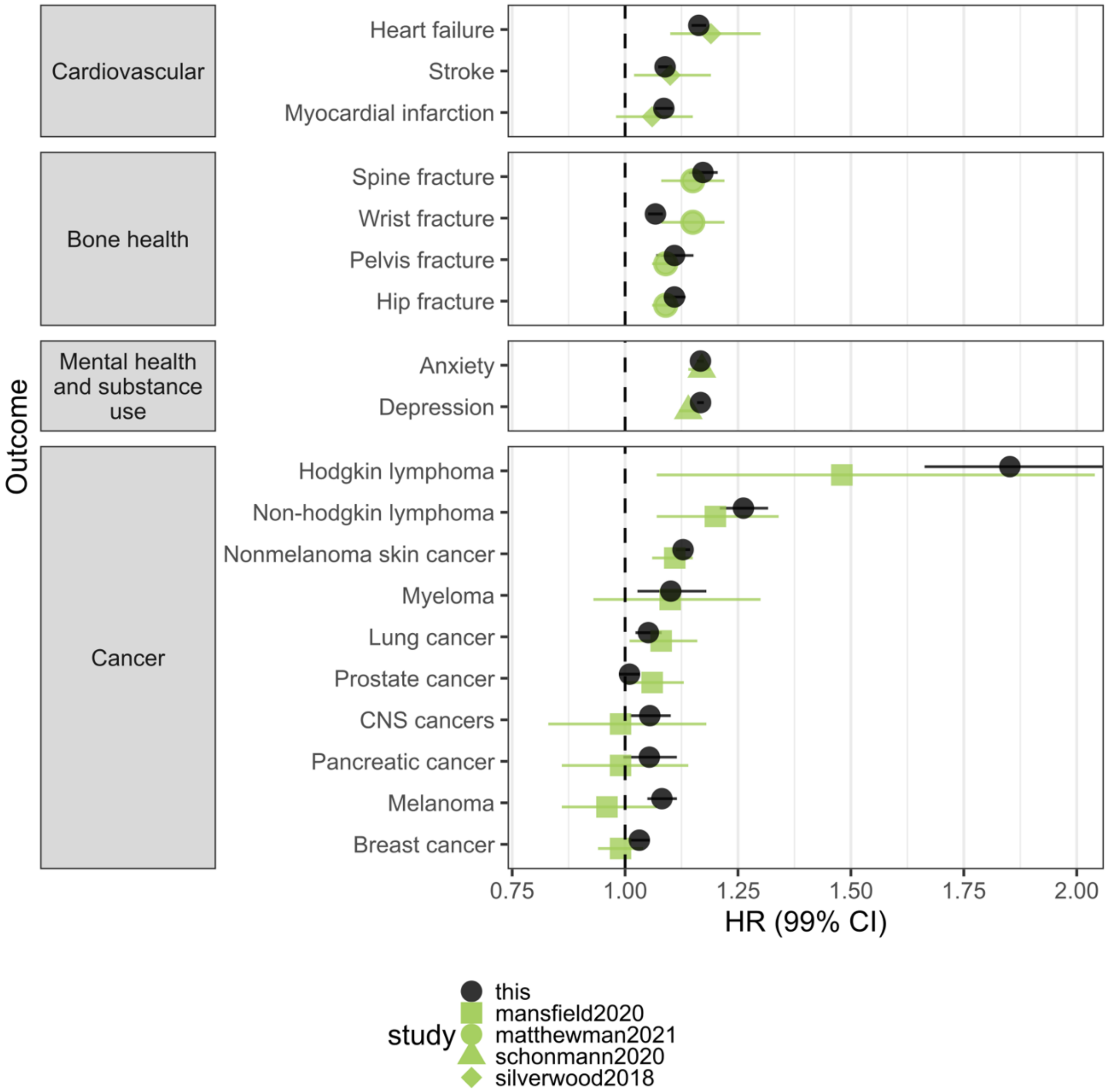

Supplementary Figure 1: Hazard ratios from Cox regression compared to results from previous studies using CPRD GOLD.
mansfield2020: Mansfield KE, Schmidt SAJ, Darvalics B, et al. Association Between Atopic Eczema and Cancer in England and Denmark. JAMA Dermatol 2020; 156: 1086.
matthewman2021: Matthewman J, Tadrous M, Mansfield KE, et al. Association of Different Prescribing Patterns for Oral Corticosteroids With Fracture Preventive Care Among Older Adults in the UK and Ontario. JAMA Dermatology 2023; 159: 961–9.
schonmann2020: Schonmann Y, Mansfield KE, Hayes JF, et al. Atopic Eczema in Adulthood and Risk of Depression and Anxiety: A Population-Based Cohort Study. The Journal of Allergy and Clinical Immunology: In Practice 2020; 8: 248-257.e16.
silverwood2018: Silverwood RJ, Mansfield KE, Mulick A, et al. Atopic eczema in adulthood and mortality: UK population-based cohort study, 1998-2016. J Allergy Clin Immunol 2021; 147: 1753–63.

Supplementary Figure 2: Comparison with previous studies (eczema severity)

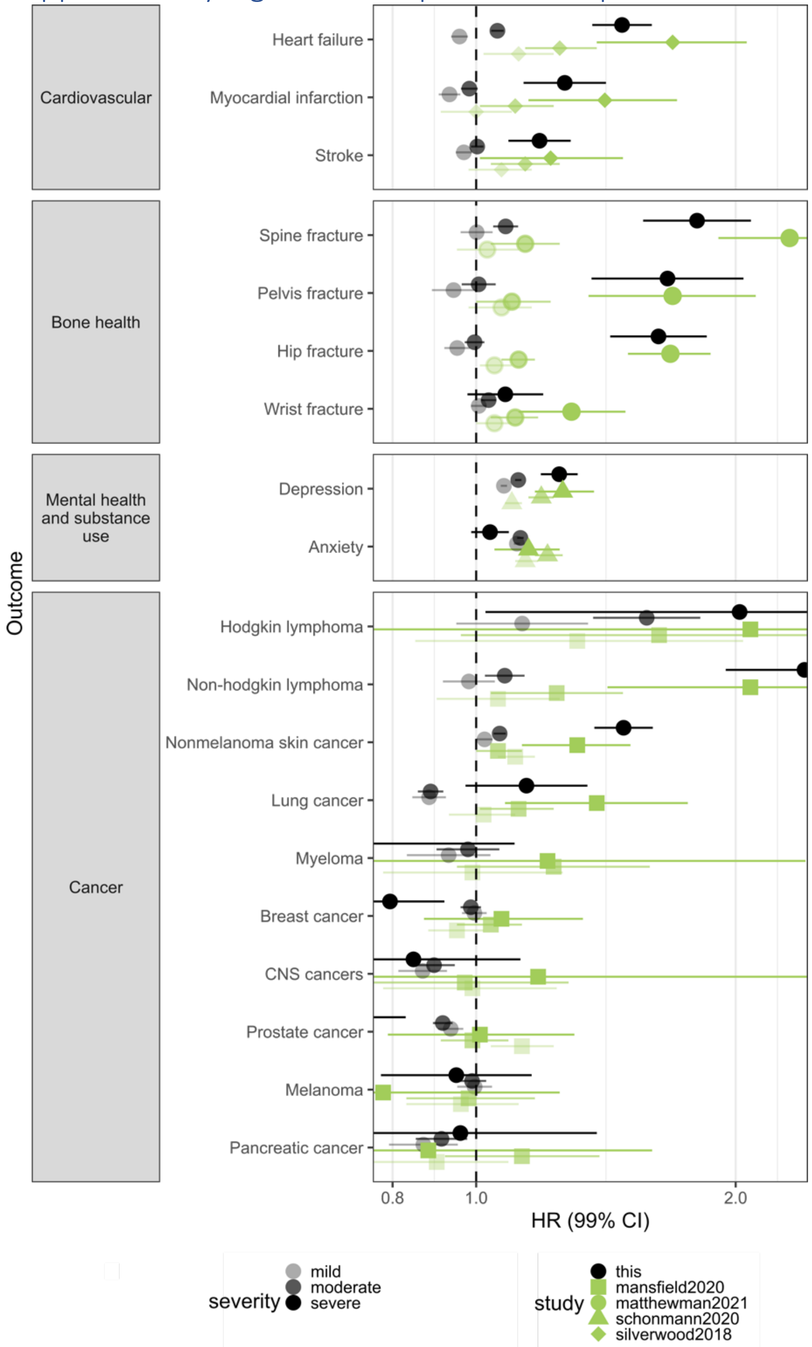

Supplementary Figure 2: Hazard ratios for mild, moderate and severe eczema from Cox regression compared to results from previous studies using CPRD GOLD. Results from this study are from comorbidity adjusted models additionally adjusted for time-updated secondary care admissions.

mansfield2020: Mansfield KE, Schmidt SAJ, Darvalics B, et al. Association Between Atopic Eczema and Cancer in England and Denmark. JAMA Dermatol 2020; 156: 1086.

matthewman2021: Matthewman J, Tadrous M, Mansfield KE, et al. Association of Different Prescribing Patterns for Oral Corticosteroids With Fracture Preventive Care Among Older Adults in the UK and Ontario. JAMA Dermatology 2023; 159: 961–9.

schonmann2020: Schonmann Y, Mansfield KE, Hayes JF, et al. Atopic Eczema in Adulthood and Risk of Depression and Anxiety: A Population-Based Cohort Study. The Journal of Allergy and Clinical Immunology: In Practice 2020; 8: 248-257.e16.

silverwood2018: Silverwood RJ, Mansfield KE, Mulick A, et al. Atopic eczema in adulthood and mortality: UK population-based cohort study, 1998-2016. J Allergy Clin Immunol 2021; 147: 1753–63

Supplementary Figure 3: Histograms of age at index date

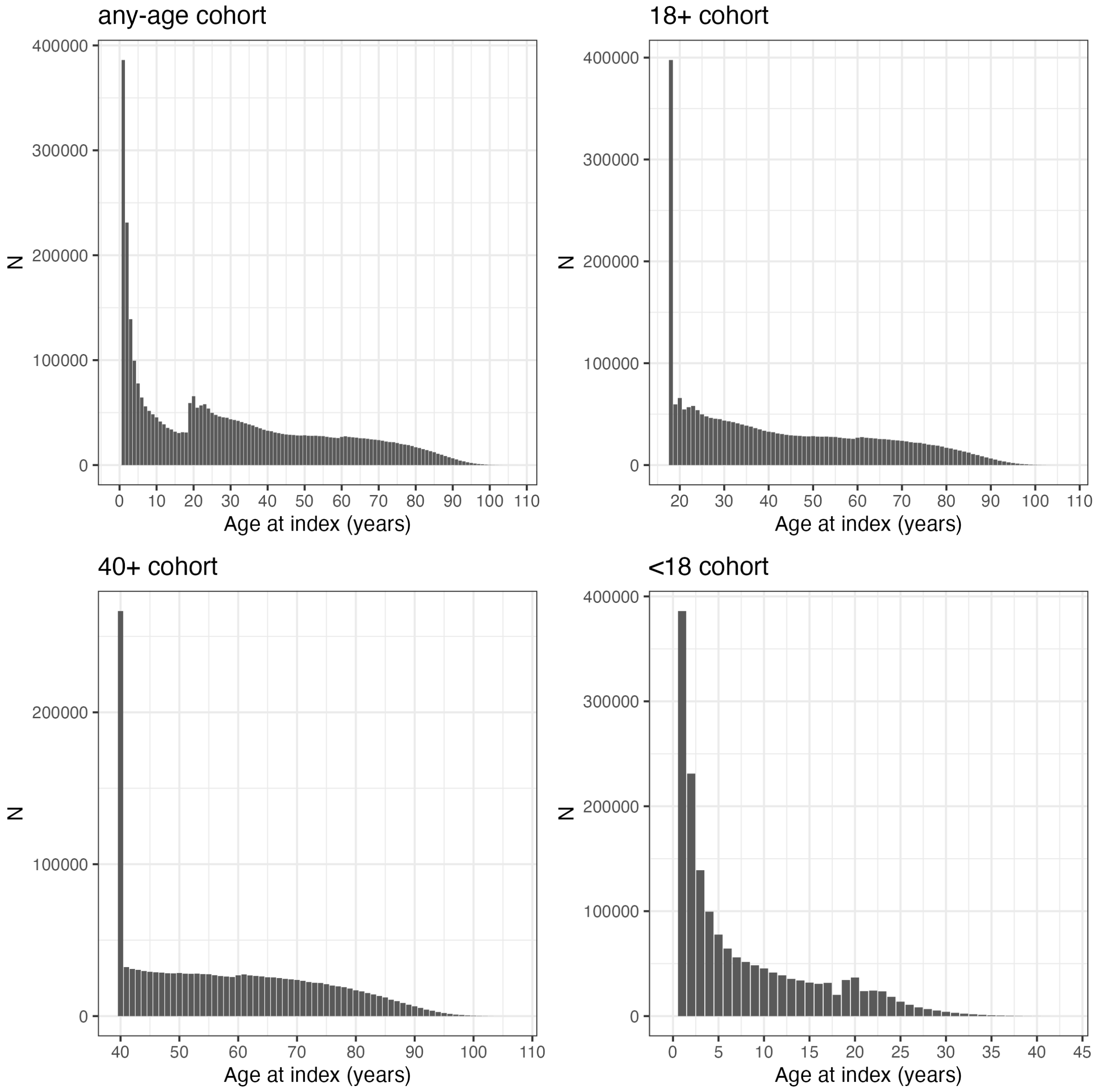

Supplementary Figure 3: Histograms showing the distribution of age at index date (in years) for the any-age cohort (top left), the 18+ cohort (top right), the 40+ cohort (bottom left) and the <18 cohort (bottom right). N denotes the number of individuals within each one-year age bin.

Supplementary Figure 4: Histograms of follow-up time

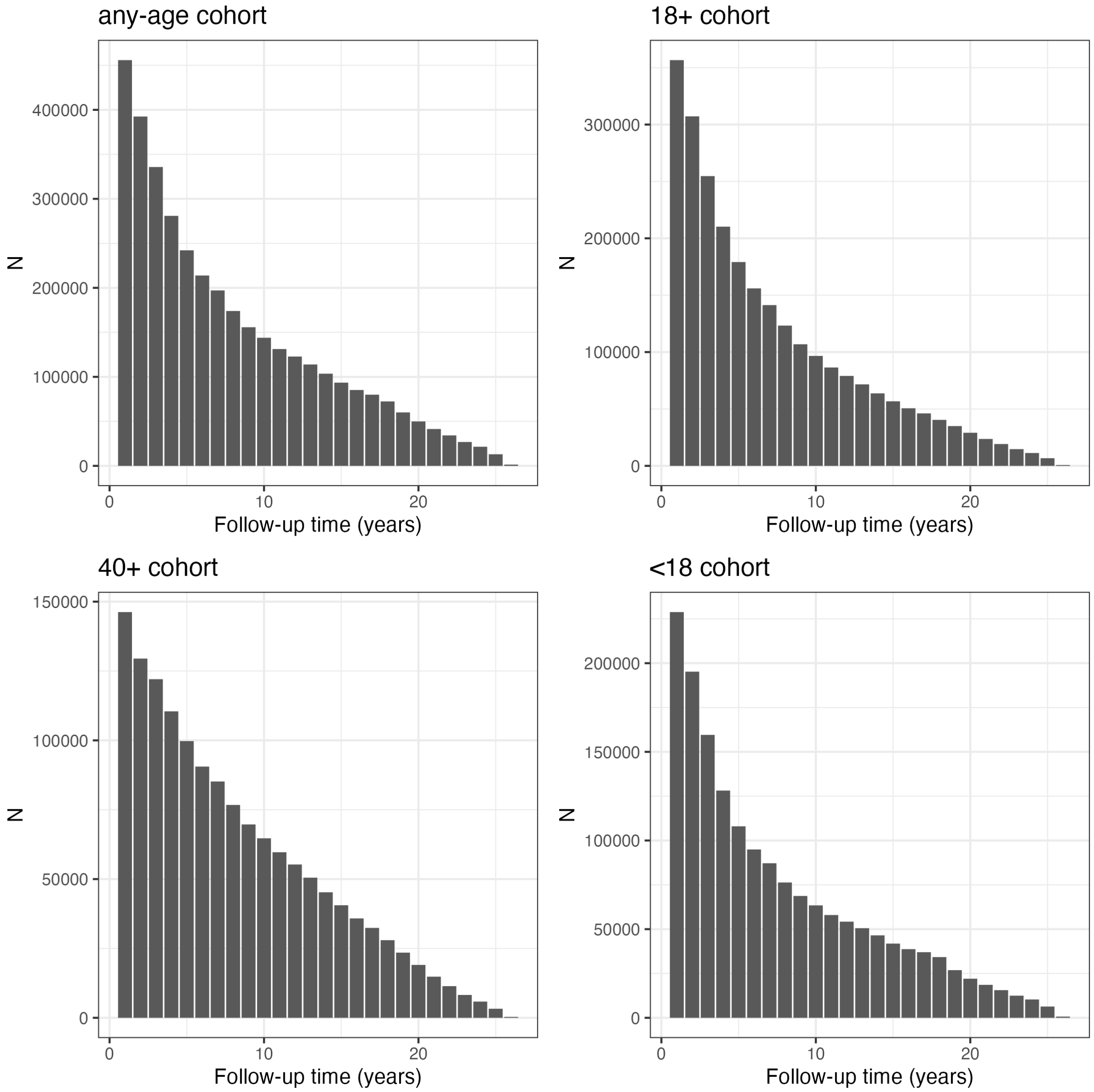

Supplementary Figure 4: Histograms showing the distribution of follow-up time (in years) for the any-age cohort (top left), the 18+ cohort (top right), the 40+ cohort (bottom left) and the <18 cohort (bottom right). N denotes the number of individuals within each one-year follow-up time bin.
